# Diversity of *Wolbachia* infections in Sri Lankan mosquitoes with a new record of *Wolbachia* Supergroup B infecting *Aedes aegypti* vector populations

**DOI:** 10.1038/s41598-024-62476-3

**Published:** 2024-05-25

**Authors:** N. D. A. D. Wijegunawardana, Y. I. N. Silva Gunawardene, W. Abeyewickreme, T. G. A. N. Chandrasena, P. Thayanukul, P. Kittayapong

**Affiliations:** 1 Graduate Program in Molecular Medicine, Faculty of Science, Mahidol University, Phayathai, Thailand; 2 Department of Bioprocess Technology, Faculty of Technology, Rajarata University of Sri Lanka, Mihintale, Sri Lanka; 3 Molecular Medicine Unit, Faculty of Medicine, University of Kelaniya, Kelaniya, Sri Lanka; 4 Department of Parasitology, Faculty of Medicine, General Sir Johan Kotelawala Defence University, Dehiwala-Mount Lavinia, Sri Lanka; 5 Department of Parasitology, Faculty of Medicine, University of Kelaniya, Kelaniya, Sri Lanka; 6 Center of Excellence for Vectors and Vector-Borne Diseases, Faculty of Science, Mahidol University, Salaya, Thailand; 7 Department of Biology, Faculty of Science, Mahidol University, Phayathai, Thailand

**Keywords:** Phylogenetics, Entomology, Bacterial evolution

## Abstract

*Wolbachia* bacteria are common endosymbionts of insects and have recently been applied for controlling arboviral vectors, especially *Aedes aegypti* mosquito populations. However, several medically important mosquito species in Sri Lanka were present with limited information for the *Wolbachia* infection status. Therefore, the screening of *Wolbachia* in indigenous mosquitoes is required prior to a successful application of *Wolbachia*-based vector control strategy. In this study, screening of 78 mosquito species collected from various parts of the country revealed that 13 species were positive for *Wolbachia* infection, giving ~ 17% infection frequency of *Wolbachia* among the Sri Lankan mosquitoes. Twelve *Wolbachia-*positive mosquito species were selected for downstream *Wolbachia* strain genotyping using Multi Locus Sequencing Type (MLST), *wsp* gene, and *16S rRNA* gene-based approaches. Results showed that these *Wolbachia* strains clustered together with the present *Wolbachia* phylogeny of world mosquito populations with some variations. Almost 90% of the mosquito populations were infected with supergroup B while the remaining were infected with supergroup A. A new record of *Wolbachia* supergroup B infection in *Ae. aegypti*, the main vectors of dengue, was highlighted. This finding was further confirmed by real-time qPCR, revealing *Wolbachia* density variations between *Ae. aegypti* and *Ae. albopictus* (*p* = 0.001), and between males and females (*p* < 0.05). The evidence of natural *Wolbachia* infections in *Ae. aegypti* populations in Sri Lanka is an extremely rare incident that has the potential to be used for arboviral vector control.

## Introduction

*Wolbachia* bacteria widely spread among varieties of insect species and exhibit different effects on the host cells or immune systems. At present, *Wolbachia* plays an important role in many of the insect vector control programs due to its biosafety and environmentally friendly nature. Its application is enormous and promising for agricultural pests such as fruit flies; flea beetle, *Aphthona* spp., to control leafy spurge^1^; house fly, *Musca domestica*, and stable fly, *Stomoxys calcitran*s^2^. In addition, this approach has been practiced for medically important vector control programs including *Aedes* vectors^3–5^; and paratransgenesis of *Wolbachia* is being considered for use in mosquitoes to reduce the spread of malaria^6^ and in tsetse fly to reduce the spread of sleeping sickness^7^. Moreover, other mosquito species, such as *Aedes polynesiensis* (South Pacific)^8^, *Aedes albopictus* (Italy)^9^, and *Culex quinquefasciatus* (southwestern Indian ocean)^10^, have also been field tested to determine the feasibility of using *Wolbachia*-based population suppression technology in the near future.

*Wolbachia* are maternally inherited and favor infected females by inducing Cytoplasmic Incompatibility (CI). The CI caused developmental failure of offspring in the cross between uninfected females and *Wolbachia*-infected males. This increased the relative success of infected females in the populations. Along with this approach, *Wolbachia* induced CI was being applied to create sexually incompatible *Ae. aegypti* male mosquitoes to be used for *Wolbachia*-based population suppression strategies, i.e., Incompatible Insect Technique (IIT) and in combined with Sterile Insect Techniques (SIT) that ionizing irradiation was to ensure the male sterility and to avoid the release of fertile female mosquitoes. On the other hand, *Wolbachia* had been implemented with the population replacement strategy, that both *Wolbachia* infected males and females were introduced in the field to establish the *Wolbachia* infected population which significantly reduced the cost of mosquito production. In fact, attention on *Wolbachia*-based approach for *Ae. aegypti* mosquito control has been drawn in Sri Lanka on both replacement and suppression strategies^11,12^. Sri Lanka released *Wolbachia*-infected mosquitoes for the pilot replacement program (20 km^2^) for dengue vector control during year 2020–2021^11^ and also research-scale trial with SIT^12^. This was supported by the present promising results recorded from several countries such as China^4^, Brazil^13^, Australia^14^, Vietnam^15^, and Thailand^16^. Therefore, there has been a strong requirement for screening of primarily major arboviral vectors and other mosquito species for autochthonous *Wolbachia* infection before implementing a large scale *Wolbachia*-infected *Ae. aegypti* release program in Sri Lanka. Identification of autochthonous *Wolbachia* infection in *Ae. aegypti* host was primarily important to reduce the risk in future failures due to generating sexually compatible mating.

In addition to CI induced by *Wolbachia*, this bacterium was also capable of inducing several other sex-related phenotypes, including male killing (MK), male feminization (MF) and thelytokous parthenogenesis (TP)^17^. An application of artificially *Wolbachia* transferring from a different host, such as *Drosophila melanogaster* into *Ae. aegypti* to suppress natural populations of dengue virus vectors, have been successfully demonstrated by many countries^18^**.** Therefore, the knowledge on *Wolbachia*-mosquito symbiosis in various autochthonous insects is a crucial factor. Identifying naturally occurring *Wolbachia* strains in mosquitoes is useful for two reasons. Firstly, the transfer of naturally occurring *Wolbachia* among closely related species is more promising than among distinct ones^19,20^, since there is an increased chance of a) not having negative effects on the host and b) successfully adapting and expressing the desired phenotype in the new host. Secondly, the dynamics of *Wolbachia* strains that are introduced into an insect population may be altered by the *Wolbachia* strains that already exist in that populations due to CI and/or competition among strains^21^.

Furthermore, besides reproductive host manipulations, *Wolbachia* can also affect nutritional and metabolic pathways of the hosts as well as can affect host development and lifespan. In addition, *Wolbachia* can provide protection of the hosts from pathogens and parasites, as well as affect host mating behavior and facilitate host speciation^17,22^. It also alters the competence of transinfected arthropod vectors for the transmission of arboviruses through competition for resources, immune-priming, induction of the phenoloxidase cascade and induction of microRNA-dependent immune pathways^22^. As *Wolbachia* provide various benefits, the application of mosquitoes control using *Wolbachia* bacteria is very promising.

*Wolbachia* autochthonous infection in *Ae. aegypti* has been recorded from time to time in several countries^18,20,23^, where the screening methods has been always doubtful either with possible contamination of the sample or false positivity due to inappropriate research practices. Therefore, our study adopted Multi Locus Sequencing Type (MLST), *wsp* gene, and *16S rRNA* gene-based approaches, together with the quantification of *Wolbachia* infection densities in different hosts through the qPCR method to confirm the detection of *Wolbachia* in *Ae. aegypti*. In addition, the investigation of *Wolbachia* diversity and density in Sri Lanka mosquitoes should be vital information for future arboviral disease control programs applying *Wolbachia* bacteria which could reduce the public health and economic burdens from mosquito-borne diseases.

## Results

### Wolbachia incidence and prevalence in Sri Lankan mosquitoes

Out of 78 mosquito species screened, 13 were positive with both *wsp* and *16S rRNA* PCR assays giving overall 16.7% infection frequency among the sampled mosquito species (Table 1). The *Wolbachia* infection frequency of *Ae. aegypti* screened was 3.35% of the wild population (17/507) and *Ae. aegypti* specimens collected from three different locations including Anuradhapura (4/107, 3.74%, collection time Oct 2014), Colombo (8/150, 5.33%, collection time June 2022), and Gampaha (5/150, 3.33%, collection time June 2022) recorded *Wolbachia* prevalence while a single infected specimen was not found in the Trincomalee sampling location (0/100, 0%, collection time June 2023) (Supplementary Table S1). For the rest of the *Wolbachia*-positive species examined, the infection rates were always 100% with 1–10 samples examined per species.

**Table 1:** *Wolbachia* prevalence in mosquitoes collected in Sri Lanka as determined by PCR amplification of *16S rRNA, wsp* and all MLST primers with assigned GenBank accession numbers.

Genus	Species/samples that were positive for at least one gene	GenBank accession numbers						
		*16S rRNA* (wspec)	*wsp*	*ftsZ*	*hcpA*	*fbpA*	*coxA*	*gatB*
*Aedes*	*Ae. aegypti*	MH447384	MH777430	MH756106	–	MH777448	MH756095	MH777437
	*Ae. albopictus*	MH447377	MH777434	MH756109	MH777464	–	MH756096	MH777438
	*Ae. pseudoalbopictus*	MH447378	–	MH756107	MH777454	–	–	MH777439
*Armigeres*	*Ae. flavus*	MH447379	–	MH756110	MH777455	MH777449	MH756097	MH777440
	*Ae. kesseli*	–	–	MH756111	MH777456	–	MH756098	MH777441
	*Ae. subalbatus*	MH447380	MH777435	MH756112	MH777457	MH777450	MH756099	MH777442
*Culex*	*Cx. fuscocephala*	–	–	MH756108	MH777458	–	MH756100	MH777443
	*Cx. gelidus*	MH447381	–	MH756113	MH777459	MH777452	MH756105	MH777447
	*Cx. pipiens*	–	MH777436	MH756114	MH777460	–	MH756103	–
	*Cx. quinquefasciatus*	MH447376	MH777431	MH756115	MH777463	MH777451	MH756104	MH777444
	*Cx. tritaeniorhynchus*	–	–	–	–	–	–	–
*Mansonia*	*Mn. indiana*	MH447382	MH777432	MH756117	MH777461	–	MH756101	MH777445
	*Mn. uniformis*	MH447383	MH777433	MH756116	MH777462	MH777453	MH756102	MH777446

### Mosquito species verification by CO I and CO II sequencing

**Figure 1:**
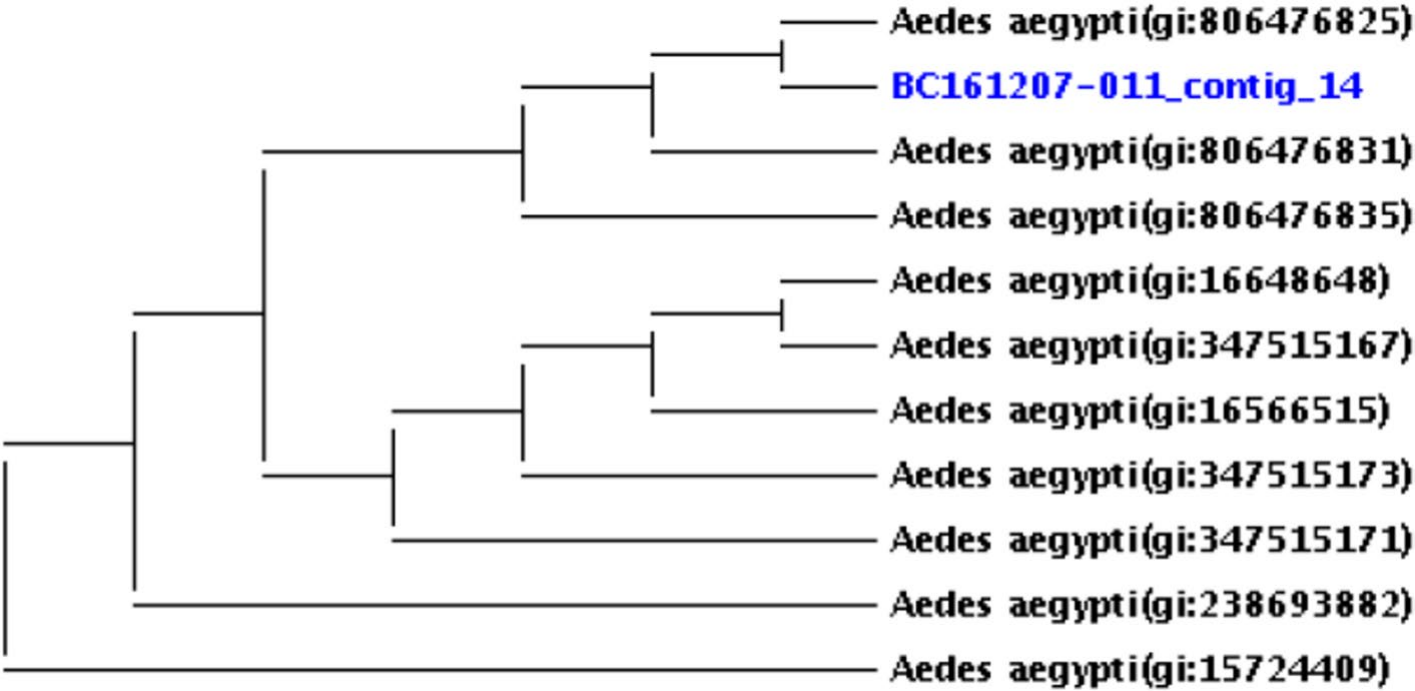
Phylogenetic tree for confirmation of the *Aedes aegypti* sample; molecular identification was performed by PCR followed by sequencing using CO I primers for *Aedes aegypti* mosquito samples. The results of phylogenetic analysis of the same samples for species confirmation (BC161207-011_contig_14) was displayed.

The result of cytochrome oxidase subunit 1 (CO I) and CO II sequencing for *Wolbachia*-positive *Aedes aegypti* species (BC161207-011_contig_14) revealed that these species, which was morphologically identified, belongs to *Ae. aegypti* (Fig. 1). The control specimens of identification based on morphological characters carried out in this study for *Ae. aegypti* were confirmed as they clustered with corresponding species in the phylogenetic analysis.

### Genotyping of the *Wolbachia* strains

**Figure 2:**
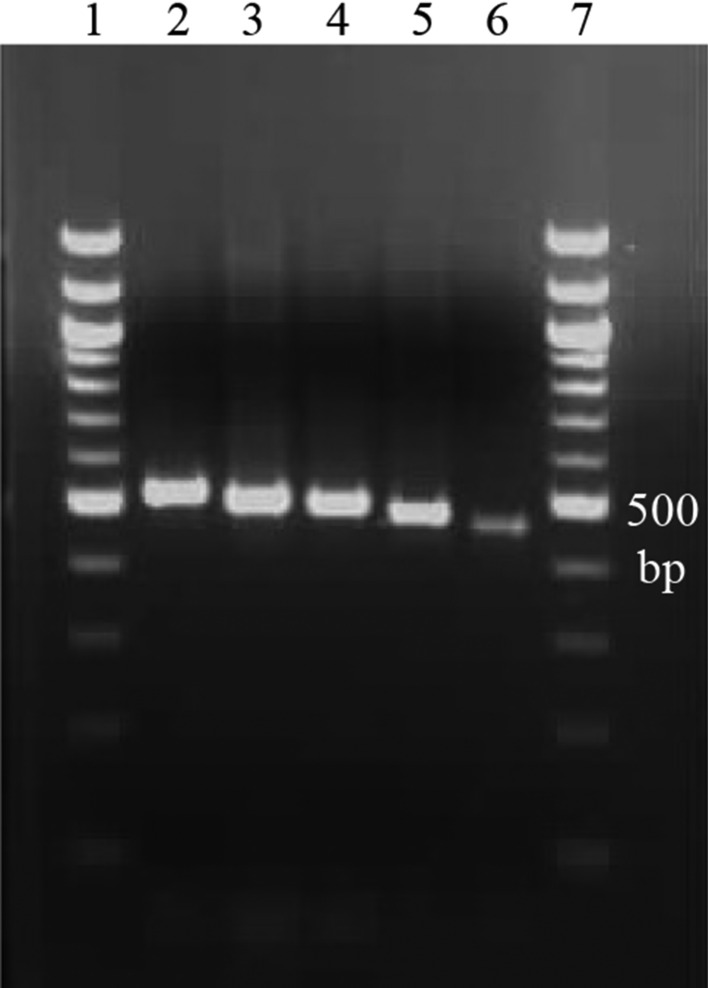
Gel image of DNA of the *Wolbachia*-infected *Mansonia indiana* sample amplified with lane 2—*ftsZ* (524 bp), lane 3—*hcpA* (515 bp), lane 4—*fbpA* (509 bp), lane 5—*coxA* (487 bp) and lane 6—*gatB* (471 bp) primers along with 100 bp marker in lane 1 and 7, respectively.

A clear single band was obtained for the DNA of 13 mosquito species with five different MLST primers (Table 2). Figure 2 described the variation of DNA band size for *Mansonia indiana* sample amplified with five different MLST primers. However, sequences obtained for the *Culex tritaeniorhynchus* sample were failed to submit to the Gene Bank database due to multiple peaks obtained. Ten species showed absence of nucleotide polymorphisms in the *wsp* sequences, suggesting that a single strain was infecting these mosquito species. The two remaining species gave multiple peaks in the chromatogram for some of the primer PCR sequences denoting possible infection of multiple strains of *Wolbachia* (results summarized in Tables 1 and 2).

**Table 2:** Allelic profiles of different *Wolbachia* strains.

Isolate	*gatB*	*coxA*	*hcpA*	*ftsZ*	*fbpA*	ST
*Ae. pseudoalbopictus*	242	NA	166	210	NA	–
*Ae. aegypti*	239	1	166	10	1	–
*Ae. albopictus*	3	2	2	10	3	2
*Ae. flavus*	239	1	269	29	412	–
*Ae. kesseli*	239	221	1	29	NA	–
*Ar. subalbatus*	239	1	269	29	412	–
*Cx. fuscocephala*	4	1	3	22	NA	–
*Cx. gelidus*	9	1	1	11	203	–
*Cx. pipiens*	4	3	3	129	NA	–
*Cx. quinquefasciatus*	239	3	269	22	4	–
*Mn. indiana*	107	87	29	35	NA	–
*Mn. uniformis*	126	14	3	73	4	–

*NA, Not available due to missing of good quality sequencing results.

### Analysis of the sequences

Polymorphism analysis for the *16S rRNA* gene revealed that a total of 55 sites analyzed with 31 invariable (monomorphic) sites and 25 polymorphic (segregating) sites. The number of positions with gaps was four and the number of positions with missing data was recorded as zero. However, the *16S* sequence attributed to *Wolbachia* from *Ae. aegypti* had highest nucleotide identity (100%) to the *Wolbachia 16S* from *Ae. aegypti* [GenBank: MF999263]. This identity was better than that of *Wolbachia 16S* sequences from the sister species within the genus *Aedes* namely *Ae. albopictus* (96%) and *Ae. pseudoalbopictus* (92%).

### Allelic profile form for different *Wolbachia* strains infecting mosquitoes

Allelic profiles of 12 different *Wolbachia* strains infecting different mosquito species were given in Table 2. Most of the alleles were exactly matched with the available alleles in the *Wolbachia* MLST database while the rest were partially matched (Table 2). All sequences obtained for *ftsZ* MLST gene perfectly matched to the existing alleles in the MLST database. However, only the *Ae. albopictus* MLST gene profile was exactly matched with the *Ae. albopictus* allelic profile already present in the *Wolbachia* MLST database (ST-2). Based on a comparison to the *Wolbachia* MLST database (http://pubmlst.org/wolbachia), 14 alleles of *gatB*, *coxA*, *hcpA* and *fbpA* were determined to be novel. The combination of alleles for all five genes in *Ae. aegypti Wolbachia* was unique and constituted a novel strain type. Most of the *Wolbachia* strain shared two or three alleles with *Wolbachia* from *closely related species or genus*.

### *Wolbachia* strain characterization based on the amino acid motifs of the hypervariable regions (HVRs) of the *wsp* sequence

Due to multiple peaks or low-quality sequences obtained in sequencing, five samples failed to sequence with *wsp* primers. Allelic profile form of rest of the samples were shown in Table 3. Accordingly, only *Ae. albopictus* and *Cx. pipiens wsp* and MLST alleles perfectly matched with the existing alleles in the database (http://pubmlst.org/wolbachia) and they were from the same mosquito host species. Other alleles partially matched with *Wolbachia* strains in the same mosquito host species or different arthropod species. Accordingly, a total of 43 new alleles were submitted to the database for allele number assignment which includes at least one allele in all the genes that were completed for both *wsp* and MLST genes.

**Table 3:** Allelic profiles of *Wolbachia* isolates based on HVRs of the *wsp* sequence.

Isolate	*wsp*	hvr1	hvr2	hvr3	hvr4
*Ae. albopictus*	**1**	**1**	**1**	**1**	**1**
*Ae. aegypti*	*408*	1	9	12	1
*Ae. kesseli*	*408*	9	9	12	86
*Cx. gelidus*	*578*	2	17	38	173
*Cx. pipiens*	**10**	**10**	**8**	11	**8**
*Cx. quinquefasciatus*	*611*	243	101	10	217
*Mn. indiana*	*584*	2	17	3	240
*Mn. uniformis*	*581*	69	88	3	23

The exact match denoted in bold letter while the partial match was given in regular font.

### Phylogenetic inferences for individual genes of *16S rRNA*, *wsp* and MLST

Based on the phylogenetic relationship between sequences in each gene, phylogenetic trees were generated separately for *16S rRNA* (Supplementary Fig. 1A), *wsp* (Supplementary Fig. 1B) and concatenated MLST sequences (Supplementary Fig. 1C). Supplementary Fig. 1-A showed the phylogenetic tree drawn from the sequence data together with reference sequences obtained from the gene bank database for the gene *16S rRNA*. The optimal tree with the sum of branch length is 0.045. Evolutionary distance between *Wolbachia* strains infected *Ae. albopictus* and *Ae. aegypti* was 0.011 with 0.005 base substitutions per site according to the above phylogeny. The *16S* sequence attributed to *Wolbachia* from *Ae. aegypti* had highest nucleotide identity (100%) to the *Wolbachia* *16S* from *Ae. aegypti* [GenBank: MF999263]. This identity was higher than that between the *Wolbachia* *16S* sequences from the sister species within the genus *Aedes* namely *Ae. albopictus* (96%) and *Ae. pseudoalbopictus* (92%).

The optimal tree with the sum of branch length was 1.413 shown in Supplementary Fig. 1-B for the phylogeny based on the *wsp* gene. According to the estimation of evolutionary distance between *Wolbachia* strains present in the mosquito host species based on the *wsp* gene sequence, there was no genetic distance between *Wolbachia* strains infecting *Ae. aegypti* and *Ae. albopictus* mosquito host species. Similarly, there was no genetic distance between *Wolbachia* strains infecting *Cx. gelidus* and *Mn. indiana* mosquito hosts. The highest evolutionary distance was obtained for the *Wolbachia* strains present in *Ae. kesseli* with both *Ae. albopictus* and *Ae. aegypti* mosquito hosts and it was indicated as 1.322.

Phylogenetic analysis of the *Wolbachia* strains of seven mosquito species, based on complete MLST gene set was given in the Supplementary Fig. 1-C. Concatenated reference sequences were also incorporated and aligned with available sequences as described earlier. *Ae. aegypti*, *Cx. gelidus*, *Mn. uniformis Wolbachia* strains cluster together while the four remaining strains (derived from *Cx. quinquefaciatus*, *Ar. subalbatus*, *Ae. flavus* and *Ae. albopictus*) clustered in a separate clade. Phylogenies using *wsp* and the concatenation of the five MLST genes showed some discrepancy in respect to the position of *Wolbachia* strains present in certain mosquito species hosts with *16S rRNA* phylogeny. Based on the *16S rRNA* and *wsp* phylogeny, *Ae. aegypti Wolbachia* was closely related to *Wolbachia* from *Ae. albopictus* (Supplementary Fig. 1- A and B) while the MLST phylogeny placed *Ae. aegypti Wolbachia* more closely related to *Wolbachia* from *Mn. uniformis* (Supplementary Fig. 1-C).

### Phylogenetic inferences for individual MLST genes

Since there were inconsistencies regarding the different datasets, not only concatenated, MLST genes were further analyzed individually. Phylogenetic tree was constructed for each aligned multiple gene sequences obtained for each of the MLST genes of the *Wolbachia* strain present in mosquitoes as assessed by sequencing of corresponding PCR products of each individual species. Phylogenetic trees were given under Supplementary Fig. 2-A—*gatB*, 2-B – *coxA*, 2-C – *hcpA*, 2-D – *ftsZ* and 2-E – *fbpA*. Numbers at the nodes of each phylogenetic tree indicated bootstrap values and reference sequences included host strains from PubMLST.

As shown in the Supplementary Fig. 2-A, the *gatB* gene-based phylogeny analysis of *Wolbachia* strains present in mosquito species showed 0.00 genetic variation between the test sequences and also with the reference sequences other than one of the *Wolbachia* B strain presents in *Ae. albopictus* from China (PubMLST allele 1760). *Wolbachia* strains present in filarial nematode (super group C) were clustered separately as expected.

According to the *coxA* gene-based phylogeny analysis of *Wolbachia* strains present in mosquito species (Supplementary Fig. 2-B), it was clear that this gene sequence was very similar or identical between *Wolbachia* strains infecting the species in one genus. Therefore, there was no or minimal genetic variation between *Wolbachia* strains infecting different host species. The *hcpA* gene-based phylogeny analysis of *Wolbachia* strains present in the mosquito hosts in Sri Lanka revealed that *Wolbachia* strains infecting *Mn. indiana* species had the *hcpA* gene sequence similar to the reference sequences of *Wolbachia* strains infecting *Cx. quinquefasciatus* (1808, 498) and *Cx. pipiens* (28) (Supplementary Fig. 2-C). Similarly, *Wolbachia* strains infecting *Mn. uniformis*, *Cx. fuscocephala* and *Cx. pipiens* had identical *hcpA* gene sequences. In addition, *ftsZ* gene sequences of *Wolbachia* strains infecting different mosquito species of the same genus were very similar (Supplementary Fig. 2-D); however, there were exceptions like in the case of the *Wolbachia* strains infecting *Cx. fuscocephala* and *Ae. pseudoalbopictus* from Sri Lanka. Similarly with other MLST genes, the *fbpA* gene-based phylogeny analysis of *Wolbachia* strains present in mosquitoes from Sri Lanka showed very close genetic structure within the same host genus (Supplementary Fig. 2-E).

**Figure 3:**
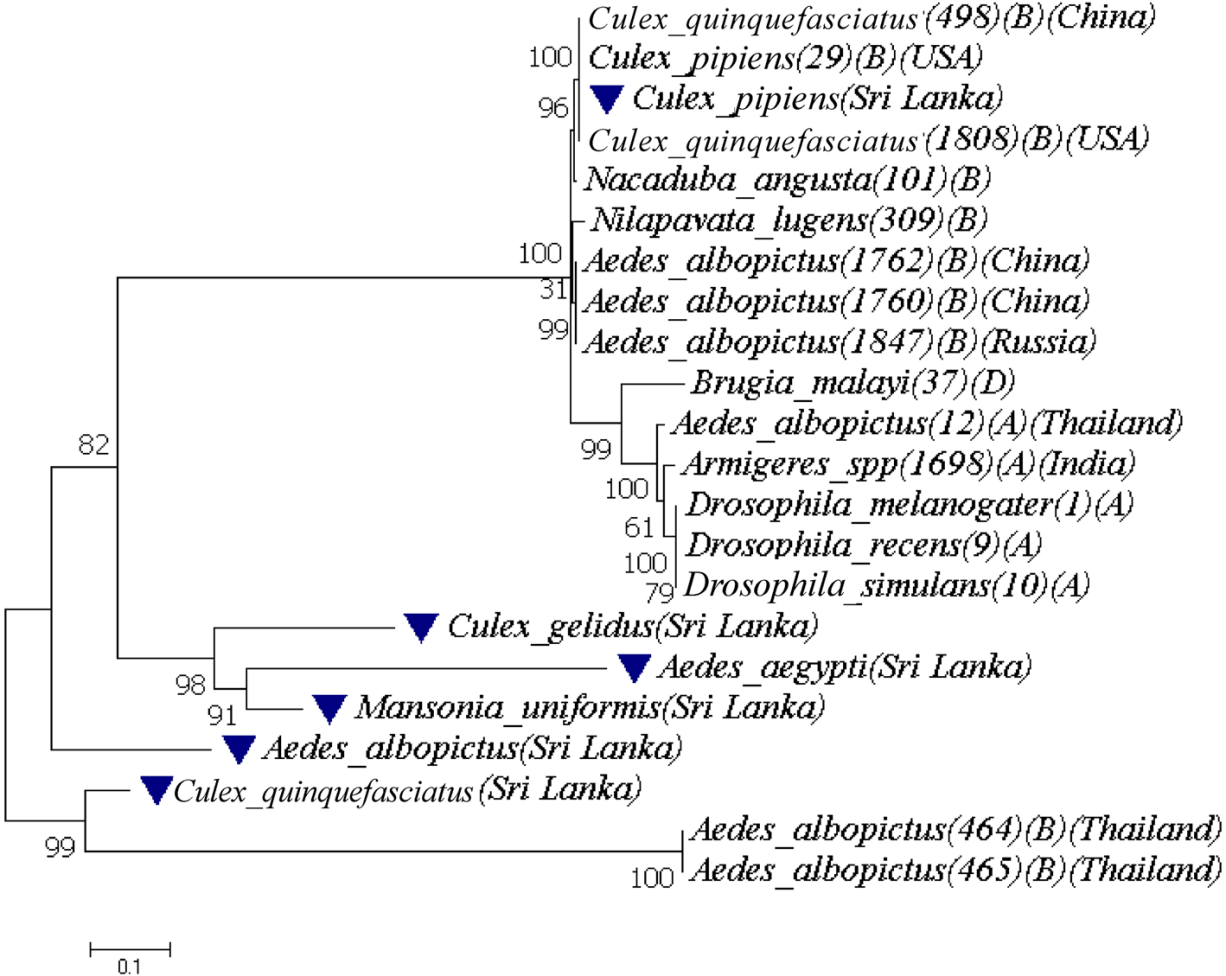
Phylogeny of *Wolbachia* strains present in the mosquito hosts from Sri Lanka based on the concatenated MLST and *wsp* genes.

All available concatenated sequences of MLST genes and *wsp* gene were again aligned and resulted concatenated sequences were aligned with reference sequences attributed in the same way. The resulting phylogenetic tree was given in Fig. 3. Out of the available complete MLST gene profile of eight *Wolbachia* strains in different mosquito hosts, only six strains had *wsp* gene sequences. Therefore, this final analysis step involved only seven *Wolbachia* isolates from different mosquito hosts, i.e., *Ae. aegypti*, *Ae. albopictus*, *Cx. gelidus*, *Cx. pipiens*, *Cx. quinquefasciatus, Mn. indiana* and *Mn. uniformis* (Table 2). According to the phylogeny of these concatenated sequences within group, the mean distance for genus *Aedes* was 0.428 and that for genus *Culex* was 0.844. The mean distance between groups were as follows: genus *Aedes* and *Culex* – 0.811; genus *Aedes* and *Mansonia* – 0.523 and genus *Culex* and *Mansonia* – 0.573. The mean diversity of the entire population was 0.706 while the highest genetic diversity was present between *Cx. pipiens* and *Ae. aegypti*. As revealed from the overall *Wolbachia* genotyping work (Fig. 3), *Ae. aegypti*, *Cx. quinquefasciatus*, *Cx. gelidus* and *Mn. uniformis* mosquito host were infected with *Wolbachia* B strain while *Cx. pipiens* infected with *w*Pip strain. Further findings proved that *Ae. albopictus* was infected with both *Wolbachia* strains A (*w*AlbA) and B (*w*Pip) supergroups. *Wolbachia* strains present in filarial nematode (*Brugia malayi*) were out rooted separately as expected.

### *Wolbachia* density in different arboviral mosquito hosts

Results of the *Wolbachia* infection dynamics in terms of Ct value of the *wsp* qPCR per insect were examined using quantitative PCR technique. Results indicated that the population of *Wolbachia* density varied between arboviral hosts and as well as depending on the host tissues. The *Wolbachia* infection density was highest in *Ae. albopictus* adult females with an average Ct value of 29.26 per insect, and it was not statistically significant between individual female *Ae. albopictus* specimens (*p* = 0.071). The lowest *Wolbachia* density was recorded for *Ae. aegypti* female mosquitos giving an average Ct value of 36.15, and the Ct value varied between 34.41 and 37.88 (limit of detection was 37.8). As the *Wolbachia* density was very low, future attempt is necessary to investigate for possible autochthonous *Wolbachia* infection in *Ae. aegypti*. In addition, the *Wolbachia* density was significantly different between individual female specimens (*p* = 0.001). *Wolbachia* density showed a gender difference, i.e., females had a higher *Wolbachia* density than males and it was also significantly different between both arboviral vectors (*Ae. aegypti* vs *Ae. albopictus*) (*p* = 0.001).

## Discussion

The presence of *Wolbachia* in mosquito species and the ability to trans-infect into the native *Wolbachia*-uninfected species and new host/strain combinations to induce reproductive and other interesting phenotypes has rendered this symbiont a promising tool to control mosquito vectors^24^. Therefore, both findings related to *Wolbachia* infection frequencies and genetic diversity were important for the success in *Wolbachia*-based population suppression strategies. The diversity of *Wolbachia* infection in mosquito taxa was reported in Southeast Asia^25,26^, Europe^27^, Africa^28^ and North America^29^. Several studies have unveiled the occurrence of natural *Wolbachia* infection in various mosquito genera, including *Mansonia*, *Aedes*, *Armigeres*, and *Culex*. While *Wolbachia* infection in *Ae. albopictus* has been frequently documented, there has been limited emphasis on the natural infection in *Ae. aegypti* mosquitoes^30^. Interestingly, despite the prevailing absence of natural *Wolbachia* infection within *Ae. aegypti* populations in most studies^25,28,31^, a recent investigation conducted by Balaji and colleagues^32^ using molecular techniques has demonstrated the presence of *Wolbachia* in *Ae. aegypti* mosquitoes collected from Coimbatore, India. Employing PCR amplification with *Wolbachia*-specific primers targeting *16S rRNA*, *wsp*, and *ftsZ* genes, this study conclusively identified *Wolbachia* supergroup B in the *Ae. aegypti* populations through phylogenetic analysis.

Notably, *Wolbachia* natural infection has been scarcely recorded in *Anopheles* and *Ae. aegypti* mosquitoes, with a few studies conducted in Malaysia^26,33^, Thailand^34,35^, Myanmar^36^, Philippines^37,38^, Panama^39^, New Mexico and Florida, USA^40^, and various regions in Africa^41–45^. A study by Wong and colleagues in Malaysia^26^ reported *Wolbachia* infection in both *Anopheles* mosquitoes (*An. balabacensis*, *An. introlatus*, *An. macarthuri*, *An. latens*, *An. maculatus*, *An. barbirostris*, *An. hyrcanus*, and *An. sinensis* amplified from *Wolbachia* specific *16S rRNA* primer) and *Ae. aegypti* (amplified from *wsp* primer).

In Sri Lanka besides from our study, there were two other studies related to frequency and distribution of *Wolbachia* within wild mosquito populations^46,47^. Nugapola and colleagues reported that they had screened a total of 330 individual mosquitoes belonging to 22 species and 7 genera, out of which 87 mosquitoes (26.36%), belonging to four species (i.e. *Ae. albopictus*, *Cx. quinquefasciatus, Ar. subalbatus* and *Mn. uniformis*), were reported as positive for *Wolbachia* natural infections as detected by *wsp* gene primers^46^. *Aedes aegypti* was negative for *Wolbachia* infection (n = 40) in which 2 samples collected from Battaramulla located around 9 km far from our Colombo study site that we observed 8 *Wolbachia*-positive *Ae. aegypti* samples among 150 samples. Another study, Tharsan et al. (2023), determined the *Wolbachia* infection in *Aedes albopictus* in Jaffna peninsula and found it widely infected with the *w*AlbA and *w*AlbB strains using *wsp* gene^47^. The gene sequence in Jaffna *Ae. albopictus* was identical to a corresponding sequence from South India but different from that in mainland Sri Lanka. Our study screened greater number of mosquitoes (n = 775) including 78 mosquito species collected from various parts of the country and revealed that 13 species were positive for *Wolbachia* infection. In addition, specific information of *Wolbachia* infection within mosquitoes in Sri Lanka was not available from the genotyping studies conducted in relation to developed MLST system. Therefore, this study focused to investigate the *Wolbachia* infections present in Sri Lankan mosquito species and genotyping the strains by several methods including *Wolbachia* specific *16S rRNA* primers, *wsp* gene based method and MLST scheme developed by Baldo and colleagues^48^ for a universal genotyping tool for *Wolbachia* which indexed variations in five conserved genes (*ftsZ*, *gatB*, *coxA*, *hcpA*, and *fbpA*).

Thus, 775 individual specimens from 78 mosquito species in Sri Lanka were screened for *Wolbachia* prior to initiation of *Wolbachia*-based *Aedes* mosquito population suppression strategy in Sri Lanka. According to PCR screening from both *16S rRNA* and *wsp* primers out of 78 species tested, 13 were positive, giving ~ 17% frequency of the prevalence of *Wolbachia* within Sri Lankan genetic background which included the genera of *Mansonia, Aedes, Armigeres* and *Culex*. This incidence estimate was compatible with all previously published estimates across arthropods. For an example a similar study conducted in a Thailand revealed that out of 89 mosquito species, the presence of *Wolbachia* was 28%^25^, consisting of the genera *Aedes, Culex, Armigeres, Coquillettidia, Hodgesia, Mansonia, Tripteroides* and *Uranotaenia.* A complete MLST profile was obtained only for 8 mosquito species and it was used for the construction of final complete phylogenetic evaluation tree. The *Wolbachia* *16S* sequence from different mosquito species had an average nucleotide identity of 96%. We used maximum likelihood to fit a beta distribution to these data to estimate the between-species distribution of prevalence. Accordingly, the *16S* phylogenetic tree resolved supergroups A to B and confirmed the identity of the sequence amplified from each mosquito that being infected with *Wolbachia* strains (Supplementary Fig. 1A). These results were further verified with both *wsp* and MLST concatenated phylogenetic trees. As observed from the sequencing results, *wsp* sequences were not identical between subspecies and had ambiguous bases. This was an indication of having multiple *Wolbachia* strains within some mosquito species. BLAST homology searches of the GenBank database confirmed strain identification as *w*Pip, the type strain associated with *Cx. pipiens*. Sequence resulted from this study was identical to the existing GenBank *wsp* sequences from California *Cx. pipiens* complex mosquitoes^25^.

In general, arthropod *Wolbachia* can be divided into a few main clades such as, A and B^48,49^, and subdivided into further strain groupings^49^. Most of these strain groups were represented in the mosquitoes and they did not show similarity with mosquito phylogenetic lineages—for example the *w*Pip subgroup occurs in several *Aedes* and *Culex* species and in an *Armigeres*^25,49^. On the other hand, superinfections of two or more *Wolbachia* strains within individuals also occurred, such as in *Ae. albopictus* where two strains from the A- and the B-clades co-existed, labelled *w*AlbA and *w*AlbB, respectively^47,50^. However, comparisons made between host and *Wolbachia* evolutionary trees strongly suggested that transfers between phylogenetically distant mosquito groups had occurred naturally^49,51,52^.

According to the maximum likelihood analysis, it revealed two major branches in the phylogenetic trees based on *Wolbachia* MLST sequences separately for each gene (Supplement Fig. 2 A-E) and collectively for all MLST genes (Fig. 1C) and *wsp* gene (Fig. 1B). These two characteristic branches clustered *Wolbachia* sequences from the studied insect host populations into two main supergroups (Fig. 1C). The first branch, which were nearly 70% of populations, harbored strains belonging to the supergroup B. The second branch included *Wolbachia*-infected populations, which suggested that approximately 30% were infected from an alternative source by strains belonging to supergroup A (e.g., *w*AlbA or *w*Ri). Additionally, this network brought more information than traditional phylogenetic tree as it showed also multiple connections among examined *Wolbachia* haplotypes (MLST strains) which could correspond to the recombination events. According to previous findings there could be a coincidental false negative sample due to *Wolbachia* tissue tropism. Nevertheless, our findings provided an estimate of the prevalence of *Wolbachia* within 78 mosquito species in Sri Lanka; and this was the first report of *Wolbachia* infection in such many mosquito species in Sri Lanka. However, consistent with previous studies done in some other countries, none of the *Anopheles* species were infected with *Wolbachia*^27,28^.

Still there are no proper documentation to prove the presence of naturally infected *Wolbachia* in the main vector of dengue (*Ae. aegypti*) and malaria (*Anopheles* spp.) disease transmission^30,31^. However, research from India recently reported *Wolbachia* infection in *Ae. aegypti* mosquito populations in the country^32^. Though there were no data on its infection frequencies and sampling population data, some of the genotyping data (*16S rRNA*) was deposited on the GenBank under the accession number MF999263. According to literature, there was no *Wolbachia-*harboring *Ae. aegypti* genomic information except that from India (*Wolbachia* MLST database allele number 1762)^32^.

In contrast to the above finding, we also found 3.35% sample frequencies of *Wolbachia* infection among *Ae. aegypti* mosquitoes collected from Gampaha District, Sri Lanka (n = 507) and were able to amplify all genes with available primers. Therefore, complete genotypic information of the *16S rRNA*, *wsp* and MLST genes were deposited on the gene bank database under the accession number MH447384. Accordingly, the evolutionary distance between *Wolbachia* strains of *Ae. albopictus* and *Ae. aegypti* was 0.011 with 0.0052 base substitutions per site based on the phylogeny of the *16S rRNA* gene. At the same time, it had the highest nucleotide identity (100%) with the *Wolbachia 16S* reference sequence of *Ae. aegypti* deposited from India [GenBank: MF999263]. This identity was better than that of *Wolbachia 16S* sequences from the sister species within the genus *Aedes* namely *Ae. albopictus* (96%) and *Ae. pseudoalbopictus* (92%). The complete allelic profile was also submitted to the *Wolbachia* MLST database.

However, autochthonous *Wolbachia* infection in *Ae. aegypti* mosquitoes always create some doubt about the data due to relatively low infection frequency and inconsistency of data^30^. Therefore, adopting several detection tools simultaneously facilitate the confirmation of the results of *Ae. aegypti* autochthonous infection with *Wolbachia*. Conversely, an argument could also be made for the possible *Wolbachia* leakage from the wild *Ae. albopictus* to wild *Ae. aegypti* mosquitoes due to back-crossing and formation of sibling species. Another possibility was the sample contamination with *Wolbachia* infected *Ae. aegypti* mosquitoes due to the release program conducted a few years back under the World Mosquito Program (WMP) in Colombo District in Sri Lanka. Even though the first argument could be accepted at certain extent since interspecific cross-mating between these two species has been documented, though until now viable offspring was not observed^53–55^. There may be a rare chance that the hybrid offspring may then mate with one of the parent species or back-cross with other hybrids, leading to further genetic and possible *Wolbachia* exchange between the two species. For the second argument, WMP released the *Wolbachia* infected mosquitoes in the Colombo Municipal Council-District 1 and Nugegoda in the years 2020–2021^11^. We detected the positive *Ae. aegypti* specimens in Anuradhapura (4/107, 3.74%, collection time Oct 2014), Colombo (8/150, 5.33%, collection time June 2022), and Gampaha (5/150, 3.33%, collection time June 2022). The collection in Anuradhapura was before the WMP released time and it was ~ 210 km far from Colombo and Nugegoda. Our Colombo and Gampaha collection sites were around 27.9 km far from the WMP released sites in CMCD1 and Nugegoda. The second argument could be doubtful because *Aedes aegypti* mosquitoes were known to have a relatively limited flight range, typically between 100 and 200 m during their lifetime. However, under certain conditions, they have been observed to travel up to 400 m or even farther. All these arguments and data suggested the necessity for more in-depth evaluation of wild *Ae. aegypti* mosquito population for possible autochthonous *Wolbachia* infection in the future.

The study conducted by the Nugapola and colleagues^46^ reported that the total of 330 individual mosquitoes, belonging to 22 species and 7 genera collected from 7 provinces in Sri Lanka, were screened for the presence of *Wolbachia* by PCR using *wsp* and *groE* primers. They found only 87 mosquitoes (26.36%) harbored *Wolbachia* which belonged to four different mosquito species namely *Ae. albopictus*, *Cx. quinquefasciatus*, *Mn. uniformis* and *Ar. subalbatus*. The results were comparable to our study. However, they have indicated that the infection frequency of *Ae. albopictus* mosquito varied between provinces while having 100% infection rate for the mosquitoes collected from the Central (n = 33) and Sabaragamuwa (n = 10) provinces followed by Southern (34.6%; 9 out of 26) and Northwestern provinces (31.0%; 9 out of 29), and only one *Ae. albopictus* was positive for *Wolbachia* from the Western province samples (3.4%; 1 out of 29)^46^. In our study, irrespective of the mosquito collection site, we observed 100% infection for all *Wolbachia*-positive mosquito species. According to the same study findings, *Cx. gelidus* collected from the Kandy District was not infected with *Wolbachia*. However, in our study, specimens collected from Colombo, Gampaha and Badulla Districts gave positive results with 100% sample infection frequency (n = 9). The *wsp* gene sequences of the *Wolbachia* strains present in *Ae. albopictus* and *Mn. uniformis* mosquito hosts clustered with the same gene locus of KY523666 and KY523674 respectively. However, *Wolbachia* strain from the *wsp* gene sequence of *Cx. quinquefasciatus* (KY523673) host was phylogenetically distinct from our results (Supplementary Fig. 1-B). Furthermore, our findings of *Ae. albopictus* superinfection with both *Wolbachia* strains belonging to A and B supergroups were in accordance with their findings of *Wolbachia* group-specific *wsp* primer PCR assays^46^. However, the GenBank submitted *Ae. albopictus wsp* sequences were reported to vary in another study conducted in different regions of Sri Lanka^47^. They had analyzed *Ae. albopictus* in Jaffna by using *wsp* primers and extensively discovered that this mosquito species harbored *w*AlbA and *w*AlbB strains of *Wolbachia* within *Ae. albopictus* population in the study location of Jaffna peninsula, Sri Lanka. They had reported that the partial gene sequence of the *w*AlbB *wsp* in Jaffna's *Ae. albopictus* matched precisely with a corresponding sequence from South India, yet it exhibited dissimilarities compared to the sequence found in mainland Sri Lanka. We found that this was an interesting finding which could lead to further investigation. In conclusion, the sample size, screening method, proper species identification, and geographic origin might be the reasons for different profiles of the mosquito species reported in these two and our studies.

## Conclusion

Infection frequencies and strain types of *Wolbachia* in mosquito species found in Sri Lankan genetic background was not significantly different from those observed in Asia or Europe, irrespective of the evidence and the presence of having *Wolbachia* in *Aedes aegypti* mosquitoes in Sri Lanka where it was not previously recorded from any countries other than India. Infection frequencies of *Wolbachia* were very low among *Ae. aegypti* mosquito populations. However, as naturally occurring vector-endosymbiont association, implying coadaptation, may have proved more stable than the artificial infection currently being used for vector control. Therefore, the evidence of natural *Wolbachia* infections in *Ae. aegypti* population in Sri Lanka is an extremely rare event which may have a potential to be used in the vector control programs. Furthermore, *Wolbachia* density as indicated by the qPCR-based methods, created new opportunities not only to determine how bacterial abundance within the arboviral mosquito populations varied but also to give the specific considerations of the specimen selection for *Wolbachia* screening.

## Materials and methods

### Collection of mosquitoes

**Figure 4:**
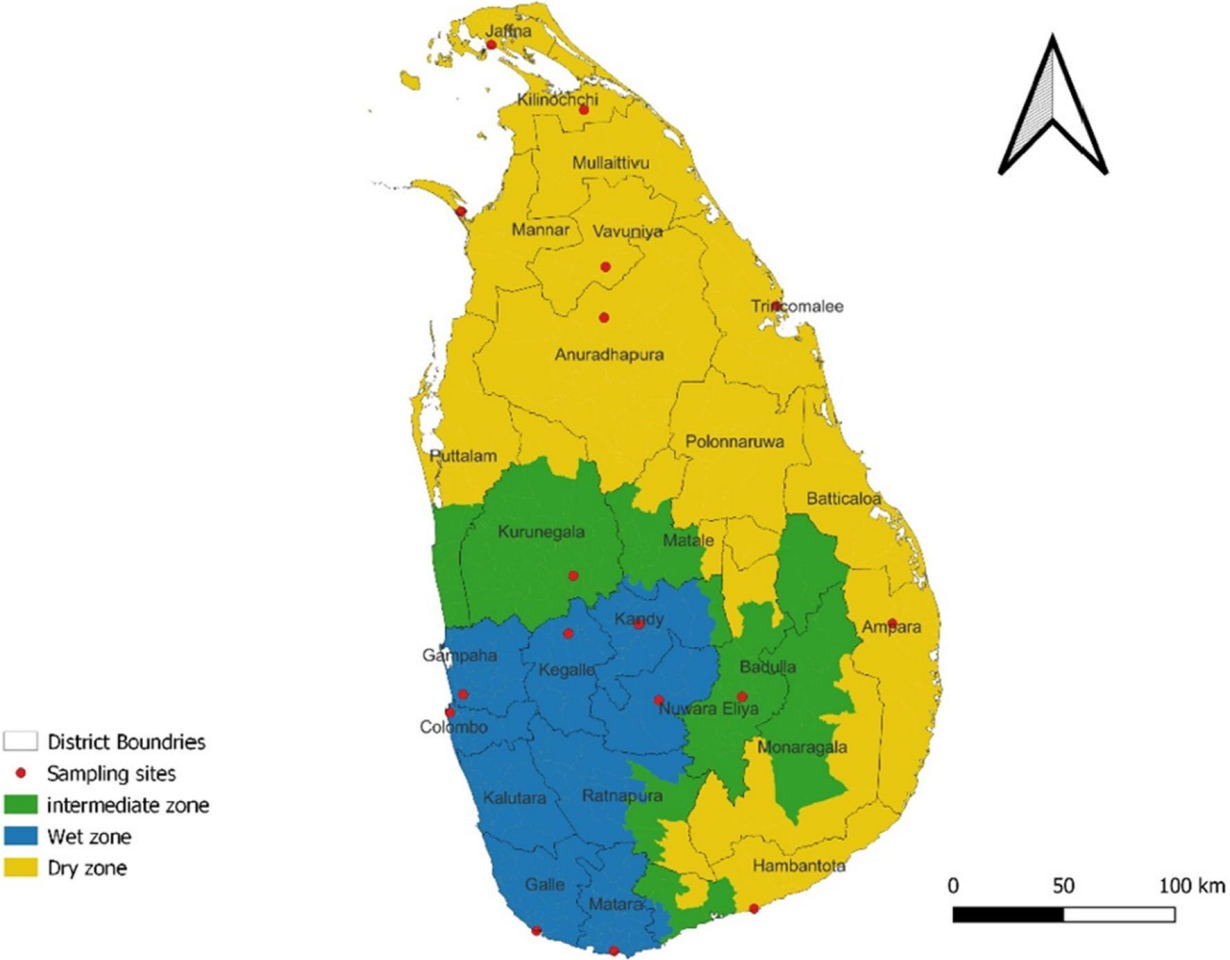
Distribution of mosquito collected sampling location in Sri Lanka; 17 locations representing the three different climatic zones (2010 – 2023). The map was created by using Qgis version 3.22 (https://download.qgis.org/qgisdata/QGIS-Website/live/html/en/site/forusers/download.html).

A total of 775 mosquito specimens were collected from western, southern, northern and central part of Sri Lanka including 16 different districts; namely Ampara (7.2917°N, 81.6726°E), Anuradhapura (8.5599°N, 80.4887°E), Badulla (6.9924°N, 81.0550°E), Colombo (6.9271°N, 79.8612°E), Galle (6.0320°N, 80.2170°E), Gampaha (6.999°N, 79.8916°E), Hambanthota (6.1237°N, 81.1034°E), Jaffna (9.6615°N, 80.0255°E), Kagalle (7.2518°N, 80.3466°E), Kandy (7.2906°N, 80.6337°E), Killinochchi (9.39487N, 80.40894E), Kurunegala (7.4840°N, 80.3666°E), Mannar (8.9769°N, 79.9022°E), Matara (5.9493°N, 80.5353°E), Nuwara Eliya (6.9785°N, 80.7133°E) and Tricomalee (8.5921°N, 81.1968°E), Vavuniya (8.7514°N, 80.4987°E) (Fig. 4). Indoor resting mosquitoes were collected using hand nets and mouth aspirators. Field mosquito collections were done using backpack aspirators, light traps, cattle baited traps, and human landing collections. After each collection step, samples were transported to the main laboratories in Sri Lanka. The animal use protocol in this study was approved by the Ethics Review Committees, Faculty of Medicine, University of Kelaniya (FWA00013225, Ref.No.P/25/03/2014); Faculty of Applied Sciences, Rajarata University (Ref: ERC/04/021); and the Animal Care and Use Committee (SCMU-ACUC), Faculty of Science, Mahidol University (Protocol No. MUSC66-031-661).

### Morphological identification of insects

Mosquitoes were sorted into morphospecies using a dissecting microscope. A “voucher” for a set of mosquitoes were taken and they were identified to species level using a set of on-line identification keys, published taxonomic keys and books^56–58^. Briefly, morphological characters present on the main body parts of adult female mosquitoes were used for the identification.

Due to the practical and cost constraint, mosquito specimen screening was limited only to female mosquitoes. The selection of female mosquito was primarily due to high *Wolbachia* density resulted for immunological staining of female mosquito tissues^57^. Female mosquitoes belonged to 7 genera, i.e., *Anopheles* (24), *Toxorhynchites* (2), *Tripteroides* (3), *Mansonia* (5), *Aedes* (16), *Armigeres* (7) and *Culex* (21) were grouped separately, and each group were divided into sub-groups according to the species. Among the 775 individual specimens collected and analyzed, 78 distinct species were identified while five specimens failed to assign to genus/species level (Supplementary Table S1). Following morphological identification, samples were stored in a freezer (-20°C) until DNA extraction.

### DNA extraction and screening of *Wolbachia* infection by *Wolbachia*-specific PCR assays

Genomic DNA was extracted from individual female mosquitoes using DNeasy Blood & Tissue Kit (Qiagen, The Netherlands) according to the protocol described by the manufacturer. To avoid the sample contamination always sample processing included negative extraction control from the known *Wolbachia* negative mosquito DNA sample isolated from the antibiotic treated 10% sugar fed *Ae. aegypti* stock caged mosquitoes maintained at the main laboratory facility in the Sri Lankan institution. Further for every PCR reaction included the known negative samples to verify the absence of contamination during the PCR master mix preparation step. PCR amplification was performed by using several different primer sets described in the Supplementary Table 2. The composition of PCR master mix and thermal profile for each primer sets were described in the Supplementary Tables 3 and 4 respectively. All PCR amplification experiments included positive and negative controls. The positive control was a *Wolbachia* double-infected *Ae. albopictus* Thailand strain sample while the negative control consisted of water as the template. Positive mosquito species for possible *Wolbachia* prevalence by both PCR assays with *16S rRNA* and *wsp* primers were noted. The DNA samples of all above positive PCR products were subjected to PCR with MLST primers to verify the *Wolbachia* strain. The MLST scheme developed by Baldo and colleagues^48^ which was used as a universal genotyping tool for *Wolbachia* which indexed variation at five conserved genes (*ftsZ*, *gatB*, *coxA*, *hcpA*, and *fbpA*) of *Wolbachia*. The PCR primers which robustly amplified loci of strains belonging to supergroups A and B and potentially amplified loci of strains belonging to other supergroups were used. A supplemental typing system developed by the same group^48^, based on the use of the hypervariable regions (HVRs) of the *Wolbachia* surface protein (*wsp*), was also used as an additional marker for strain typing. Importantly, *wsp* typing could be complement the MLST information as it was analogous to antigen protein typing used for pathogenic bacteria^59^**.** Each PCR amplification process underwent three replicates to validate the results obtained. A fourth screening was performed for selected individual samples that had conflicting results based on the above three prior replicates. Therefore, the criteria set in reporting the certainty for *Wolbachia* infection was based on at least two successful amplifications of the molecular markers. Due to the absence of certain morphological identification keys for some specimens, such specific samples were PCR amplified and sequenced with Cytochrome Oxidase I (CO I) and CO II primers (Supplementary Table S2). For confirmation and validation of the sequencing results, two DNA samples from *Ae. albopictus* and *Ar. subalbatus* were also amplified with CO I and CO II primers and sequenced.

### Sequencing, sequence alignments and assemblies

The PCR products were purified using either Montage PCR centrifugal filter devices (Millipore, USA) or QIAquick 96 PCR Purification kit (Qiagen, The Netherlands) and bi-directionally sequenced. Amplified PCR products from each molecular marker were sent for sequencing to Eurofins, Operon – Japan. An internal fragment of each PCR product was specifically selected for MLST. Forward and reverse sequences from each PCR product were aligned and visually inspected using both SeqManII by DNAStar and BioEdit DNA Sequence Analysis Software version 7.0.9 (Ibis BioSciences, USA). The contig sequence obtained from aligning both forward and reverse sequences were used for BLAST search of the nucleotide reference sequences on PubMed. Then contig was align with reference sequences and phylogenetic tree was constructed for each contig separately to verify the mosquito species with CO I and CO II PCR products and other primer PCR products for *Wolbachia* strain identification. In this circumstance, consensus sequences obtained from each individual for each gene were aligned and compared; and all sequence differences between *Wolbachia* strains were checked to confirm whether they had unambiguous peaks. As bacteria from each mosquito species had the same sequences, a consensus sequence for each gene per mosquito host species was obtained. All consensus sequences were trimmed to the appropriate length for database query. Finally, a BLAST search was performed for each sequence in the *Wolbachia* MLST database (http://pubmlst.org/wolbachia). Where a sequence had an exact match in the database, it was assigned the designated allele number. The complete MLST profiles were submitted to the *Wolbachia* MLST database and have been assigned the ID numbers (http://pubmlst.org/wolbachia). Similarly, the *Wolbachia*-positive *16S rRNA* gene PCR products were directly sequenced, and the resulted sequences were compared with the available data in the GenBank database (www.ncbi.nlm.nih.gov) using BLAST search. The above sequences were also deposited in the GenBank under assigned accession numbers provided by the GenBank.

### Phylogenetic analysis

All *Wolbachia* gene sequences generated in this study were manually edited with SeqManII by DNAStar (Version 11.1) and aligned using MUSCLE and ClustalW, as implemented in Geneious 5.3.4, and adjusted by eye. Phylogenetic analyses were performed using Bayesian Inference (BI) and Maximum-Likelihood (ML) estimation for a concatenated data set of the protein-coding genes (*gatB*, *fbpA*, *hcpA*, *ftsZ* and *coxA*) and for *wsp* and *16S rRNA* separately. For the Bayesian inference of phylogeny, PAUP version 4.0b10 was used to select the optimal evolution model by critically evaluating the selected parameters using the Akaike Information Criterion. Finally, *wsp*, *Wolbachia* specific *16S rRNA* and MLST gene forward and reverse sequences were used to reconstruct the contig file for each gene and for each species. Then all contig files were used to do BLAST nucleotide search for the reference sequences and based on the analysis and BLAST search results, the phylogenetic tree was constructed.

Algorithm used for phylogenetic reconstruction was PHASE. The evolutionary history was inferred using the Maximum-Likelihood (ML) method. The optimal tree with the sum of branch length was obtained for all primer pairs individually and for the respective concatenated sequences. The percentage of replicate trees in which the associated taxa clustered together in the bootstrap test (1000 replicates) were always maintained more than 75 bootstrap values. The evolutionary distance between *Wolbachia* strains infecting in each mosquito species were measured based on the base substitutions per site.

### Real-time quantitative PCR for *Wolbachia* density in mosquito

To estimate *Wolbachia* densities, a real-time quantitative PCR assay based on a single-copy gene *wsp* encoding a surface protein of *Wolbachia* was used to determine *Wolbachia* density in the arboviral hosts including *Ae. aegypti* and *Ae. albopictus*. Primers were specifically designed to detect the *Wolbachia* strain and amplified 332-bp to 513-bp regions of the *wsp* gene (Supplementary Table S2). The amplification reaction was monitored using a set of fluorescent probes specific to the PCR product. PCR was performed under the following conditions: 10 min at 55 °C, 1 min at 95 °C, followed by 40 cycles of 95 °C for 30 s and 60 °C for 30 s, followed by 95 °C for 1 min, 55 °C for 30 s and 95 °C for 30 s. The PCR in a 10 μL reaction system was well-optimized with 5 μL of qPCR dye, the 0.25 μL from 10 μmol/L concentration of each primer (F/R), 1 μL of template DNA, and 3.5 μL of DNase/RNase-free water. *Wolbachia* density was compared according to Ct values against the threshold value of 0.2.

## Conflict of interest

The authors have no competing interests to declare that are relevant to the content of this article.

### Supplementary Information

Supplementary Information.

## Data Availability

All data generated or analyzed during this study are included in this published article and its supplementary information files.
