# Supplementary material for: Diversity of Wolbachia infections in Sri Lankan mosquitoes with a new record of Wolbachia Supergroup B infecting Aedes aegypti vector populations
Source: Sci Rep. 2024 May 25;14:11966. doi: 10.1038/s41598-024-62476-3 (PMC11127934; doi:10.1038/s41598-024-62476-3)
Supplement: Supplementary file 1 — Supplementary Information. [file 41598_2024_62476_MOESM1_ESM.pdf]

Supplementary Materials

Diversity of *Wolbachia* infections in Sri Lankan mosquitoes with a new record of *Wolbachia* Supergroup B infecting *Aedes aegypti* vector populations

N.D.A.D. Wijegunawardana, Y.I.N. Silva Gunawardene, W. Abeyewickreme , T.G.A.N. Chandrasena, P. Thayanukul, and P. Kittayapong

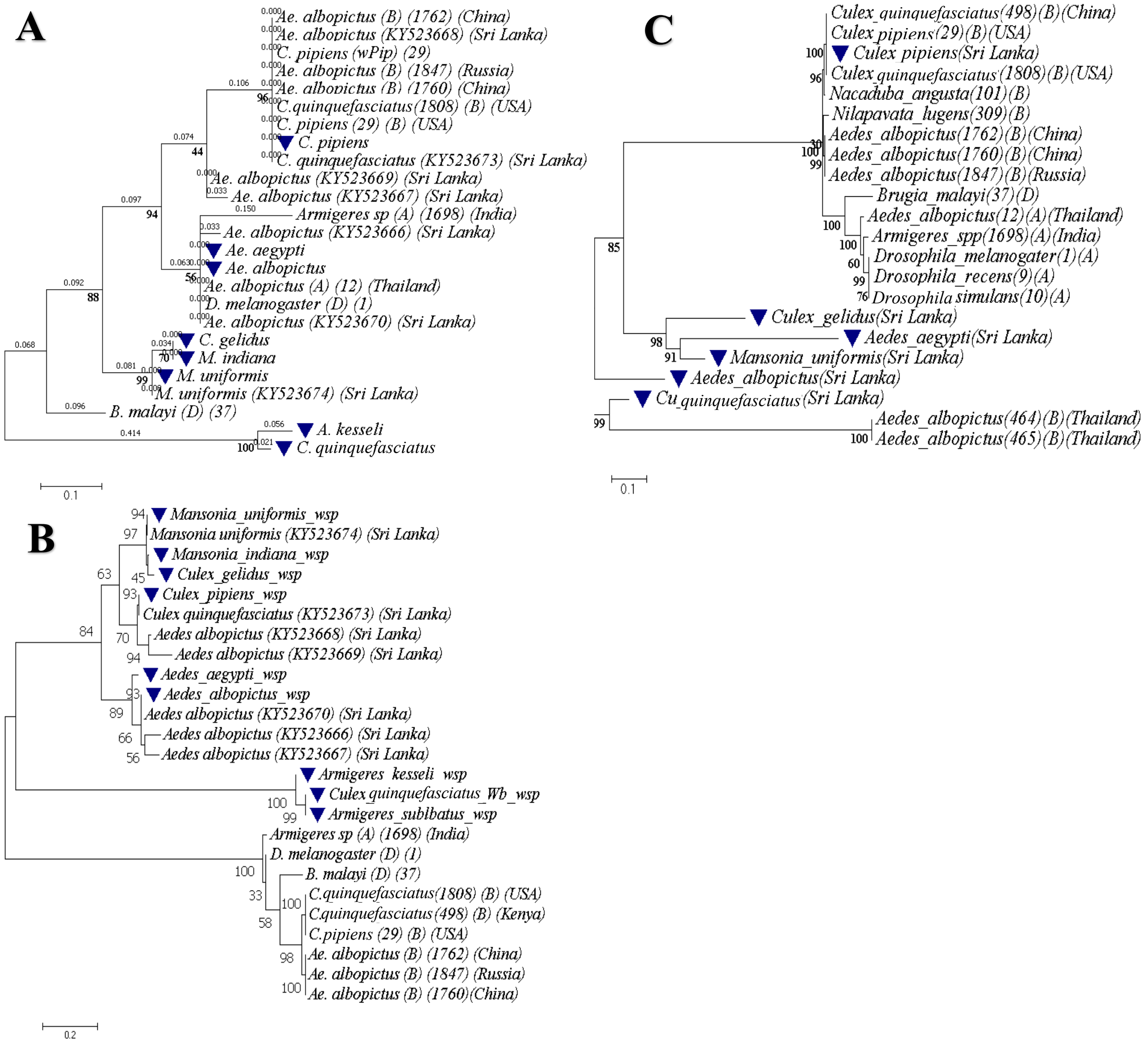

Supplementary Figure 1: *Wolbachia* phylogenetic tree drawn from the sequence data together with reference sequences obtained from the GenBank database. (A) 16S rRNA phylogeny (1 kb), (B) *wsp* phylogeny (590 bp), and (C) phylogeny based on the concatenation of *gatB*, *coxA*, *hcpA*, *ftsZ* and *fbpA* genes (2,074 bp). The evolutionary history was inferred by using the Maximum Likelihood method based on the Tamura-Nei model. The *Wolbachia* D group (allele 37) is used as an out group. *Wolbachia* strains are indicated by their host names and strain names when available. Numbers at the nodes indicate bootstrap values. Only bootstraps higher than 60 are shown. Species are abbreviated as follows; i.e., *Aedes* species: *Ae. aegypti, Ae. albopictus*, *Ae. pseudoalbopictus*; *Armigeres* species *Ar. flavus*, *Ar. subalbatus*, *Ar. kesseli*; *Culex* species: *Cx. gelidus*, *Cx. pipiens*, *Cx. quinquefasciatus*; *Mansonia* species: *Mn. indiana*, *Mn. uniformis*; *Brugia malayi*; and *Drosophila melanogaster*.

2A

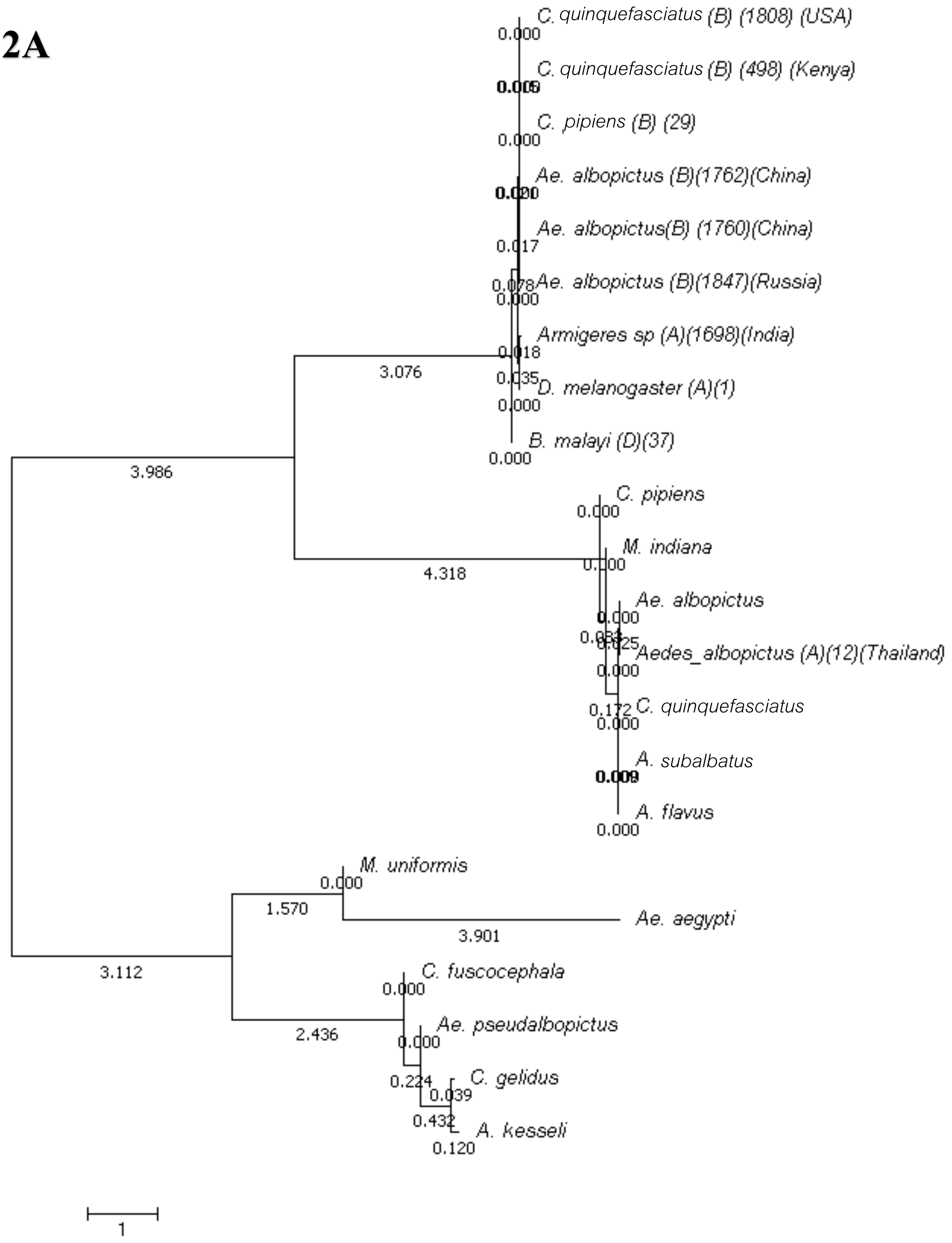

C. quinquefasciatus (B) (1808) (USA)
C. quinquefasciatus (B) (498) (Kenya)
C. pipiens (B) (29)
Ae. albopictus (B)(1762)(China)
Ae. albopictus(B) (1760)(China)
Ae. albopictus (B)(1847)(Russia)
Armigeres sp (A)(1698)(India)
D. melanogaster (A)(1)
B. malayi (D)(37)
C. pipiens
M. indiana
Ae. albopictus
Aedes_albopictus (A)(12)(Thailand)
C. quinquefasciatus
A. subalbatus
A. flavus
M. uniformis
Ae. aegypti
C. fuscocephala
Ae. pseudalbopictus
C. gelidus
A. kesseli
3.076
3.986
4.318
1.570
3.901
3.112
2.436
0.224
0.432
0.120
0.172
1

2B

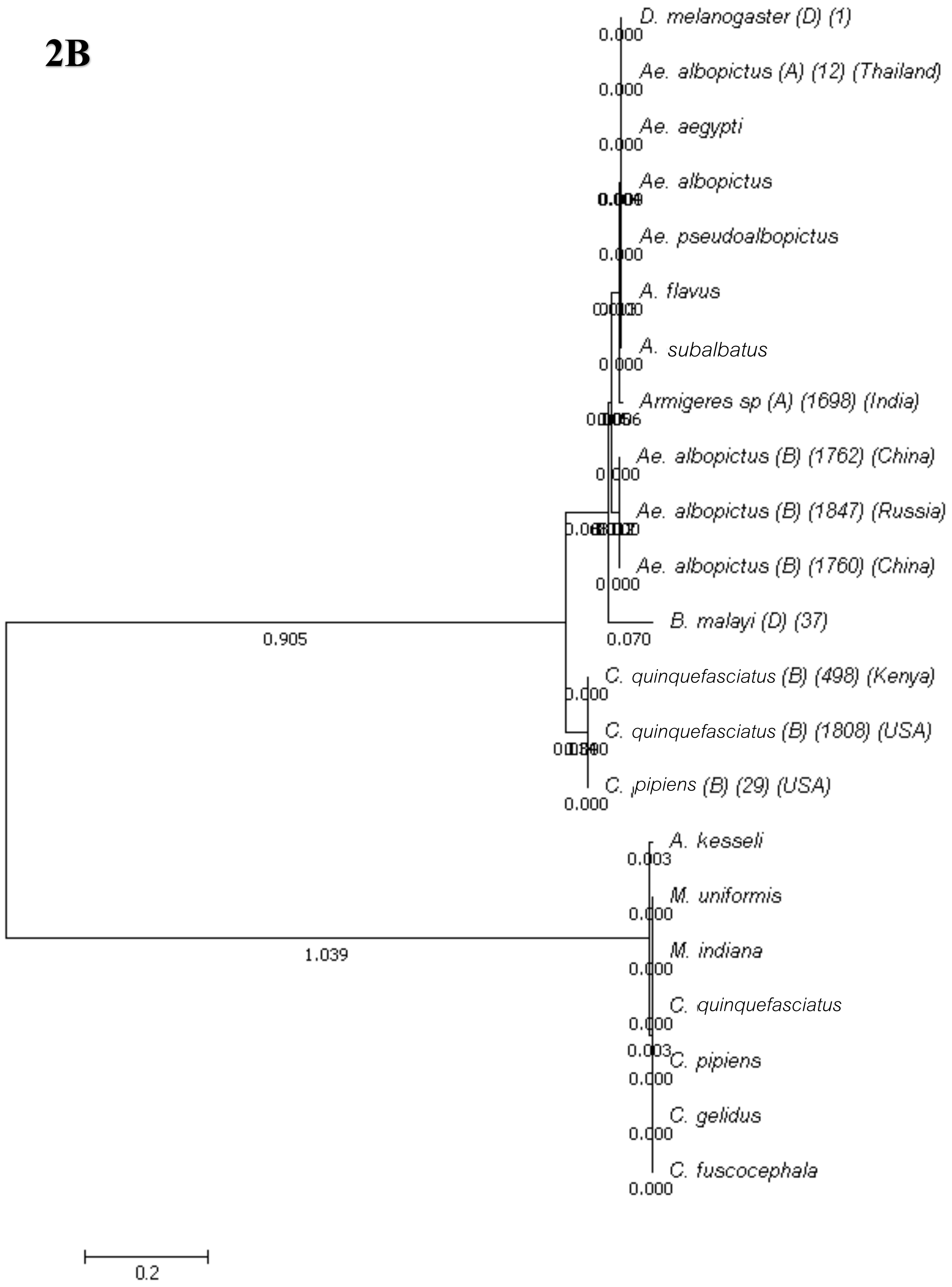
D. melanogaster (D) (1)
Ae. albopictus (A) (12) (Thailand)
Ae. aegypti
Ae. albopictus
Ae. pseudoalbopictus
A. flavus
A. subalbatus
Armigeres sp (A) (1698) (India)
Ae. albopictus (B) (1762) (China)
Ae. albopictus (B) (1847) (Russia)
Ae. albopictus (B) (1760) (China)
B. malayi (D) (37)
C. quinquefasciatus (B) (498) (Kenya)
C. quinquefasciatus (B) (1808) (USA)
C. pipiens (B) (29) (USA)
A. kesseli
M. uniformis
M. indiana
C. quinquefasciatus
C. pipiens
C. gelidus
C. fuscocephala
0.905
1.039
0.070
0.003
0.2

2C

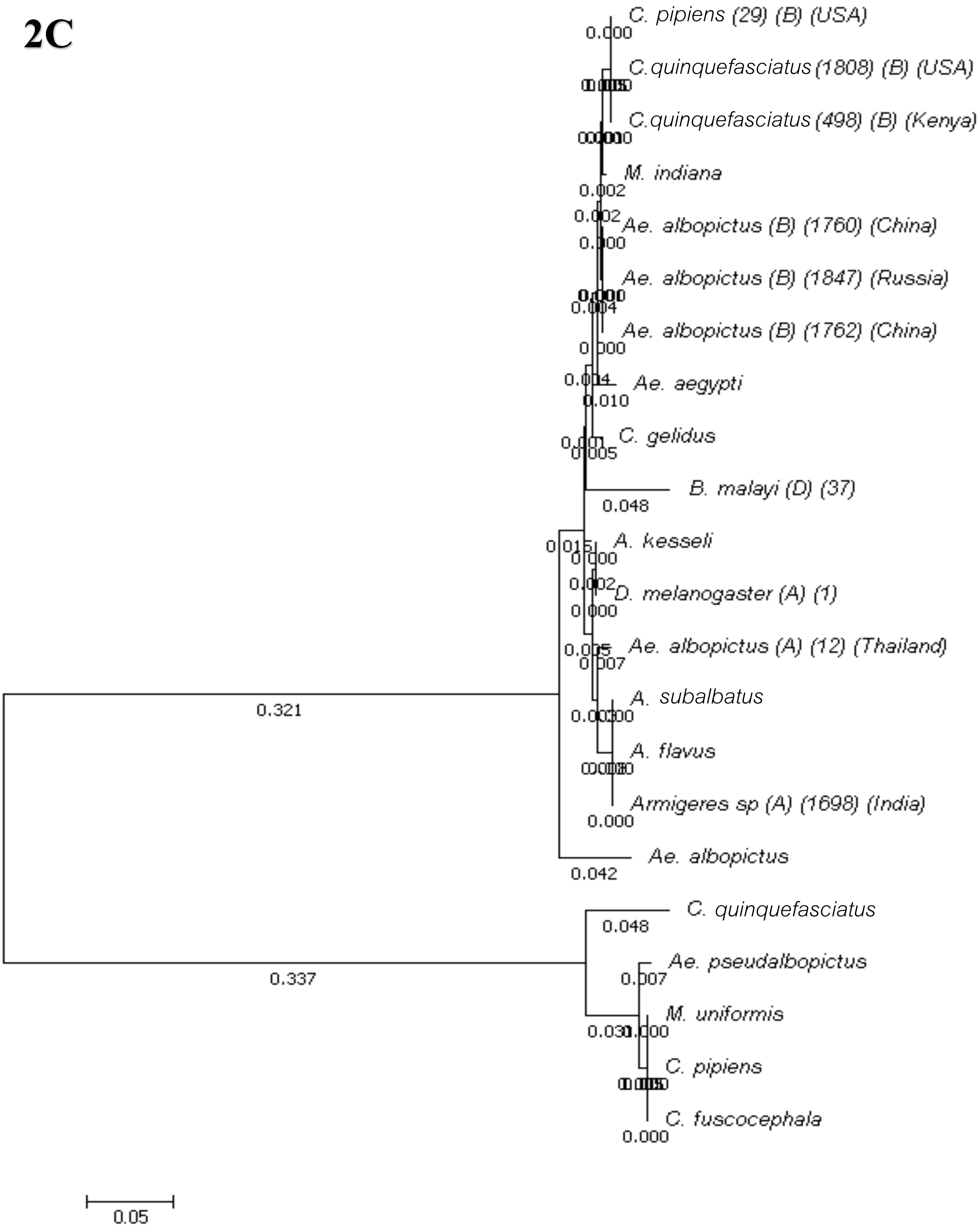
C. pipiens (29) (B) (USA)
C.quinquefasciatus (1808) (B) (USA)
C.quinquefasciatus (498) (B) (Kenya)
M. indiana
Ae. albopictus (B) (1760) (China)
Ae. albopictus (B) (1847) (Russia)
Ae. albopictus (B) (1762) (China)
Ae. aegypti
C. gelidus
B. malayi (D) (37)
A. kesseli
D. melanogaster (A) (1)
Ae. albopictus (A) (12) (Thailand)
A. subalbatus
A. flavus
Armigeres sp (A) (1698) (India)
Ae. albopictus
C. quinquefasciatus
Ae. pseudalbopictus
M. uniformis
C. pipiens
C. fuscocephala
0.321
0.337
0.048
0.042
0.05

2D

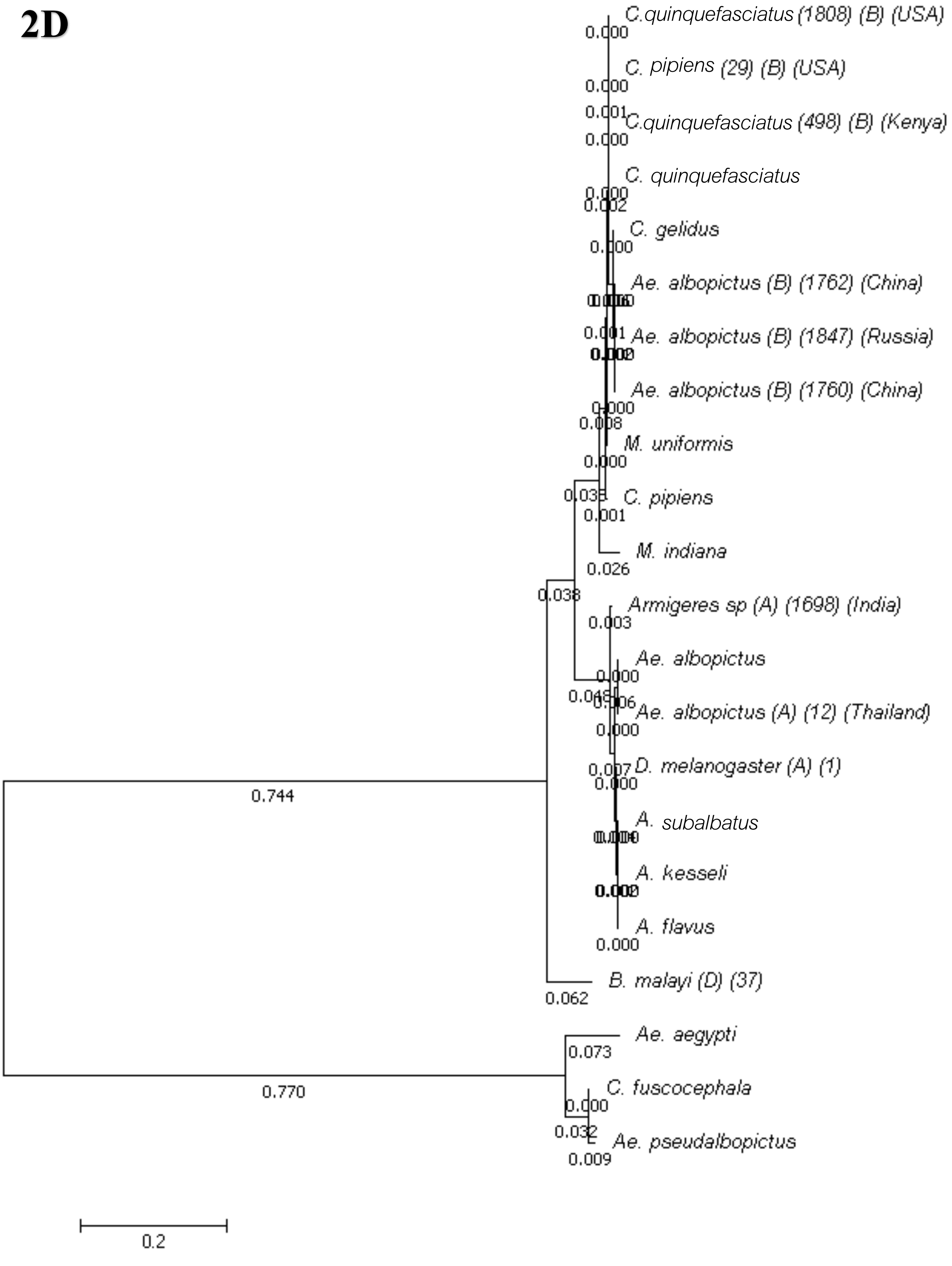

C.quinquefasciatus (1808) (B) (USA)
C. pipiens (29) (B) (USA)
C.quinquefasciatus (498) (B) (Kenya)
C. quinquefasciatus
C. gelidus
Ae. albopictus (B) (1762) (China)
Ae. albopictus (B) (1847) (Russia)
Ae. albopictus (B) (1760) (China)
M. uniformis
C. pipiens
M. indiana
0.026
Armigeres sp (A) (1698) (India)
0.003
Ae. albopictus
Ae. albopictus (A) (12) (Thailand)
D. melanogaster (A) (1)
A. subalbatus
A. kesseli
A. flavus
B. malayi (D) (37)
0.062
0.744
Ae. aegypti
0.073
C. fuscocephala
Ae. pseudalbopictus
0.009
0.770
0.2

2E

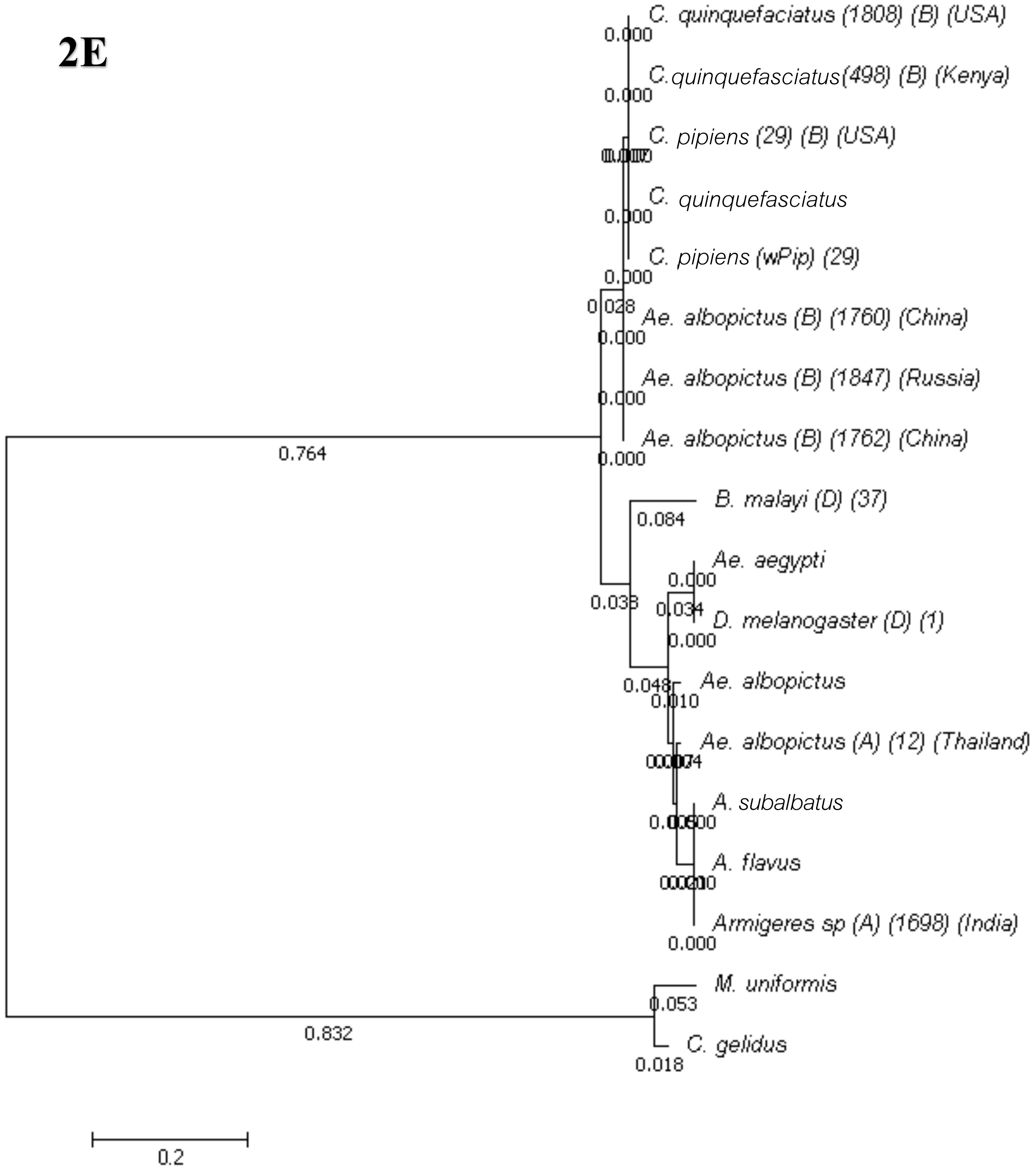

Supplementary Figure 2: Phylogenetic trees analysis of *Wolbachia* strains present in the mosquito species in Sri Lanka, based on different MLST genes: (A) *gatB*, (B) *coxA*, (C) *hcpA*, (D) *ftsZ* and (E) *fbpA*. Numbers at the nodes of each phylogenetic tree indicate bootstrap values. Reference sequences included the host strains from PubMLST.

Supplementary Table S1. Mosquito species screened for *Wolbachia* with *wsp* and *Wolbachia*-specific 16S rRNA primers along with location of collection, collection date, and initial sample size screened.

Genus	Species	Collection location (District)	Collection date (mm/yy)	Sample size
Aedes (16 species)	¶*Aedes (Stegomyia) aegypti* Linnaeus, 1762	¶Anuradhapura, ¶Colombo, ¶Gampaha, Trincomalee	Oct-14 Jun-22 Jun-23	507
	¶*Aedes (Stg) albopictus* Skuse, 1894	¶Anuradhapura, ¶Gampaha, ¶Colombo, ¶Kandy	Nov-14 Jun-22 Jun-23	21
	Aedes (Finlaya) albotaeniatus Theobald, 1908	Trincomalee	Oct-14	4
	Aedes (Stg) craggi Barraud, 1923	Kandy, Badulla	Nov-14	5
	Aedes (Aedimorphus) jamesi Edwards, 1914	Ampara	Nov-14	4
	Aedes (Stg) krombeini Huang, 1975	Kandy	Nov-14	5
	Aedes (Fin) niveus Ludlow, 1903	Kandy	Nov-14	4
	Aedes (Fin) novoniveus Barraud, 1934	Kandy, Badulla	Nov-14	4
	Aedes (Aed) pallidostriatus Theobald, 1907	Hambanthota, Matara	Dec-13	3
	Aedes (Stg) perplexus Leicester, 1908	Kandy, Badulla	Dec-13	5
	Aedes (Stg) pseudalbopictus Borel, 1928	Kandy, Badulla	Nov-14	6
	Aedes (Cancraedes) simplex Theobald, 1903	Gampaha, Kandy	Mar-14	3
	Aedes spp.	Hambanthota	Dec-13	2
	Aedes (Verrallina) uniformis Theobald, 1910	Anuradhapura, Jaffna, Trincomalee	Sep-13 Jun-22 Jun-23	12
	Aedes (Stg) vittatus Bigot, 1861	Kandy	Dec-13	5
	Aedes (Aed) vexans Meigen, 1830	Hambanthota	Dec-13	5
Anopheles (24 species)	*Anopheles (Cellia) aconitus* Donitz, 1902	Anuradhapura, Trincomalee, Killinochchi	Feb-14 Jun-22 Jun-23	8
	Anopheles (Anopheles) aitkenii James, 1903	Anuradhapura, Kandy	Oct-14 Jun-22 Jun-23	10
	Anopheles (Cel) annularis Van der Wulp, 1884	Anuradhapura, Trincomalee, Killinochchi	Feb-14 Jun-22 Jun-23	10
	Anopheles (Ano) barbirostris Van der Wulp, 1884	Anuradhapura, Nuwara Eliya, Trincomalee, Mannar, Killinochchi	May-14 Jun-22 Jun-23	10
	Anopheles (Ano) barbumbrosus Strickland & Choudhury, 1927	Anuradhapura, Trincomalee, Mannar	Nov-13 Jun-22 Jun-23	10

Genus	Species	Collection location (District)	Collection date (mm/yy)	Sample size
	Anopheles (Cel) culicifacies Giles, 1901	Anuradhapura, Kurunegala, Nuwara Eliya, Trincomalee	Sep-14 Jun-22 Jun-23	10
	Anopheles (Cel) elegans James, 1903	Kandy	Oct-14	5
	Anopheles (Ano) gigas refutans Alcock, 1913	Nuwara Eliya, Kandy	Sep-14	5
	Anopheles (Ano) insulaeflorum Swellengrebel & Swellengrebel de Graff, 1919	Nuwara Eliya, Kandy	Aug-14	5
	Anopheles (Ano) interruptus Puri, 1929	Nuwara Eliya, Kandy	Aug-14	5
	Anopheles (Cel) jamsii Theobald, 1901	Anuradhapura, Trincomalee, Mannar, Killinochchi	Mar-14 Jun-22 Jun-23	10
	Anopheles (Cel) jeyporiensis James, 1902	Anuradhapura, Trincomalee	Oct-13 Jun-22 Jun-23	10
	Anopheles (Cel) kawari James, 1903	Trincomalee, Kandy	Feb-14	4
	Anopheles (Cel) latens Sallum & Peyton, 2005	Kandy	Oct-14	4
	Anopheles (Cel) maculatus maculatus Theobald, 1901	Kurunegala, Nuwara Eliya, Kandy	Oct-14	4
	Anopheles (Ano) nigerrimus Giles, 1900	Anuradhapura, Trincomalee, Mannar, Killinochchi	Jan-14 Jun-22 Jun-23	10
	Anopheles (Cel) pallidus Theobald, 1901	Anuradhapura, Trincomalee, Mannar, Killinochchi	Jan-14 Jun-22 Jun-23	10
	Anopheles (Ano) peditaeniatus Leicester, 1908	Trincomalee, Mannar, Killinochchi	Nov-13	5
	Anopheles (Ano) peytoni Kulasekera Harrison & Amerasinghe, 1989	Kandy, Trincomalee	Oct-14	5
	Anopheles (Cel) pseudojamesi Strickland & Chowdhury, 1927	Trincomalee, Mannar, Killinochchi	Feb-14	4
	Anopheles (Cel) subpictus Grassi, 1899	Anuradhapura, Trincomalee, Mannar, Killinochchi	Feb-14 Jun-22 Jun-23	10
	Anopheles (Cel) tessellatus Theobald, 1901	Anuradhapura, Trincomalee, Killinochchi	Nov-13 Jun-22 Jun-23	10
	Anopheles (Cel) vagas vagus Donitz, 1902	Kurunegala, Nuwara Eliya, Trincomalee, Mannar, Killinochchi	Mar-14	5
	Anopheles (Cel) varuna Iyengar, 1924	Anuradhapura, Kurunegala, Kandy, Trincomalee, Mannar, Killinochchi	Mar-14 Jun-22 Jun-23	10
Armigeres (7 species)	*Armigeres (Arm) aureolineatus* Leicester, 1908	Anuradhapura, Kandy	Nov-14 Jun-22 Jun-23	10

Genus	Species	Collection location (District)	Collection date (mm/yy)	Sample size
	Armigeres (Lei) balteatus Macdonald, 1960	Badulla	Oct-14	4
	¶*Armigeres (Lei) flavus* Leicester, 1908	¶Anuradhapura, ¶Gampaha	Oct-14<br>Jun-22<br>Jun-23	10
	Armigeres kesseli Ramalingam, 1987	Gampaha	Oct-14	5
	Armigeres (Leicesteria) magnus Theobald, 1908	Gampaha	Oct-14	5
	Armigeres (Lei) omissus Edwards, 1914	Gampaha	Oct-14	5
	¶*Armigeres (Armigeres) subalbatus* Coquillett, 1898	¶Anuradhapura, ¶Gampaha, ¶Colombo, ¶Galle	Oct-14<br>Jun-22<br>Jun-23	10
Culex (21 species)	*Culex (Culiciomyia) bahri* Edwards, 1914	Nuwara Eliya, Badulla	Oct-14	4
	Culex (Cux) bitaeniorhynchus Giles, 1901	Gampaha, Colombo	Nov-14	3
	Culex (Eumelanomyia) brevipalpis Giles, 1902	Gampaha	Dec-13	4
	Culex (Lutzia) fuscanus Wiedemann, 1820	Kandy	Nov-14	4
	¶*Culex (Cux) fuscocephala* Theobald, 1907	¶Kandy, ¶Badulla	Nov-14	5
	¶*Culex (Cux) gelidus* Theobald, 1901	¶Trincomalee, ¶Gampaha, ¶Colombo	Dec-13	5
	Culex (Cux) hutchinsoni Barraud, 1924	Nuwara Eliya	Dec-13	4
	Culex (Eum) malayi Leicester, 1908	Matara, Galle	Oct-14	4
	Culex (Cux) mimulus Edwards, 1915	Kandy	Oct-14	3
	¶*Culex (Neoculex) pipiens* Linnaeus, 1758	¶Gampaha, ¶Colombo, ¶Galle	Dec-13	5
	Culex (Lasiosiphon) pluvialis Barraud, 1924	Gampaha	Dec-13	4
	Culex (Cux) pseudovishnui Colless, 1957	Kandy, Badulla	Dec-13	5
	¶*Culex (Cux) quinquefasciatus* Say, 1823	¶Anuradhapura, ¶Gampaha, ¶Colombo, ¶Galle	Oct-13<br>Jun-22<br>Jun-23	10
	Culex (Culex) sinensis Theobald, 1903	Matara, Galle	Mar-14	5
	Culex (Cux) sitiens Weidemann, 1828	Matara, Galle	Oct-14	4
	Culex spp.	Badulla	Mar-14	2
	Culex spp.	Trincomalee	Nov-14	2
	¶*Culex (Cux) tritaeniorhynchus* Giles, 1901	¶Anuradhapura, ¶Trincomalee, ¶Hambanthota	Nov-14<br>Jun-22<br>Jun-23	10

Genus	Species	Collection location (District)	Collection date (mm/yy)	Sample size
	Culex (Lophoceraomyia) uniformis Theobald, 1905	Matara, Galle	Oct-14	5
	Culex (Cux) vishnui Theobald, 1901	Badulla, Nuwara Eliya	Sep-13	5
	Culex (Cux) whitmorei Giles, 1904	Kandy, Gampaha, Colombo	Oct-14	5
Mansonia (5 species)	*Mansonia (Mansonioides) annulifera* Theobald, 1901	Anuradhapura, Trincomalee	Dec-13<br>Jun-22<br>Jun-23	10
	Mansonia (Mnd) dives Van der Wulp, 1881	Nuwara Eliya, Kegalla	Dec-13	4
	¶*Mansonia (Mnd) indiana* Edwards, 1930	¶Anuradhapura,<br>¶Trincomalee	Dec-13<br>Jun-22<br>Jun-23	10
	Mansonia spp.	Trincomalee	Nov-13	3
	¶*Mansonia (Mnd) uniformis* Theobald, 1901	¶Anuradhapura,<br>¶Gampaha,<br>¶Trincomalee,<br>¶Colombo	Oct-14<br>Jun-22<br>Jun-23	10
Toxorhynchites (2 species)	*Toxorhynchites (Toxorhynchites) minimus* Theobald, 1905	Gampaha	Nov-14	5
	Toxorhynchites (Tox) splendens Wiedemann, 1819	Gampaha	Nov-14	5
Tripteroides (3 species)	*Tripteroides (Tripteroides) affinis* Edwards, 1913	Kandy, Kegalla, Badulla, Nuwara Eliya	Nov-14	5
	Tripteroides (Rachionotomyia) aranoides Theobald, 1901	Kegalla, Badulla, Nuwara Eliya	Nov-14	5
	Tripteroides (Trp) ceylonensis Theobald, 1905	Kegalla, Badulla, Nuwara Eliya	Dec-13	5

¶ *Wolbachia*-infected species and locations

Supplementary Table S2. PCR primers used for mosquito screening of *Wolbachia* bacterial DNA

Gene	Product	Primer		Expected amplicon size	Reference of the primers
		**Designation**	**Sequence (5'–3')**		
CO I	Mitochondrial gene cytochrome oxidase c subunit I	*CO I*-F	GGATTTGGAAATTGATTAGTTCCTT	735 bp	Chan et al., 2014
		CO I-R	AAAAATTTTAATTCCAGTTGGAACAG C		
CO II	Mitochondrial gene cytochrome oxidase c subunit II	A-tLEU - F	AATATGGCAGATTAGTGCA		Liu & Beckenbach, 1992
		A-tLEU - R	GTTTAAGAGACCAGTACTT	690 bp	
glt	*Wolbachia* genes encoding citrate synthase	W*glt*AF1	TAC GAT CCA GGG TTT GTT TCT AC	639 bp	Augustinos et al., 2011;
		W*glt*ARev1	CTC ATT AGC TCC ACC GTG TG		
***gro*EL**	Heat-shock protein 60	W*gro*F1	GGTGAGCAGTTGCAAGAAGC	630 bp	Augustinos et al., 2011; Wiwatanaratanabutr et al., 2009
		W*gro*Rev1	AGATCTTCCATCTTGATTCC		
16S rRNA	Partial small subunit ribosomal DNA	16S-F (*WspecF)*	AGCTTCGAGTGAAACCAATTC	1 kb	Baldo *et al*, 2006; O'Neill *et al*., 1992; Foster *et al*., 2008
		16S-R (*WspecR)*	GAAGATAATGACGGTACTCAC		
**MLST**					
coxA	Cytochrome *c* oxidase subunit I	*cox*A_F1	TTGGRGCRATYAACTTTATAG	487 bp	Baldo *et al*, 2006
		*cox*A_R1	CTAAAGACTTTKACRCCAGT		
fbpA	Fructose-bisphosphate aldolase	*fbp*A_F1	GCTGCTCCRCTTGGYWTGAT	509 bp	Baldo *et al*, 2006
		*fbp*A_R1	CCRCCAGARAAAAYYACTATTC		
ftsZ	Cell division protein	*fts*Z_F1	ATYATGGARCATATAAARGATAG	524 bp	Baldo *et al*, 2006
		*fts*Z_R1	TCRAGYAATGGATTRGATAT		
gatB	Glutamyl-tRNA (Gln) amidotransferase, subunit B	*gat*B_F1	GAKTTAAAYCGYGCAGGBGTT	471 bp	Baldo *et al*, 2006
		*gat*B_R1	TGGYAAYTCRGGYAAAGATGA		
hcpA	Conserved hypothetical protein	*hcp*A_F1	GAAATARCAGTTGCTGCAAA	515 bp	Baldo *et al*, 2006
		*hcp*A_R1	GAAAGTYRAGCAAGYTCTG		
wsp	Outer surface coat protein gene	*wsp*_F1	GTCCAATARSTGATGARGAAAC	546 bp	Baldo *et al*, 2006; Zhou et al., 1998; Armbruster et al., 2003
		*wsp*_R1	CYGCACCAAYAGYRCTRTAAA		

Supplementary Table S3. Components required for the preparation of PCR master mix.

Reagent	(1 X) sample				
	**16S rDNA**	**wsp**	**MLST**	**glt/groEL**	**CO I/II**
Water	12.0 µl	14.4 µl	12.2 µl	14.4 µl	17.2 µl
Taqman buffer (10X)	2.0µl	2.0 µl	2.0 µl	2.0 µl	2.5 µl
$MgCl_2$ (25 mM)	2 µl	0.5 µl	1.2 µl	0.5 µl	1.0 µl
dNTP mix (10 mM)	1 µl	0.5 µl	0.4 µl	0.5 µl	0.25 µl
Forward primer (10 mM)	0.5 µl	0.2 µl	1.0 µl	0.2 µl	0.35 µl
Reverse primer (10 mM)	0.5 µl	0.2 µl	1.0 µl	0.2 µl	0.35 µl
Taq DNA polymerase (5U/µl)	1 µl	0.2 µl	0.2 µl	0.2 µl	0.25 µl
DNA sample	1 µl	2 µl	2 µl	2 µl	1 µl
**Total**	**20 µl**	**20 µl**	**20 µl**	**20 µl**	**25 µl**

Supplementary Table S4. Thermal profiles for each primer PCR program.

Parameters / Primer	Initial denaturation	Denaturation	Annealing	Extension	Final extension	Final hold
CO I / CO II	94°C, 3 min	94°C, 1 min	51°C, 1 min	72°C, 2 min	72°C, 10 min	4°C ≅
		35 cycles				
***Glt / gro*EL**	95°C, 3 min	94°C, 1 min	48°C, 1 min	72°C, 1 min	72°C, 10 min	4°C ≅
		30 cycles				
16S rDNA	94°C, 3 min	94°C, 1 min	55°C, 1 min	72°C, 2 min	72°C, 10 min	4°C ≅
		35 cycles				
wsp	95°C, 5 min	95°C, 30 sec	59°C, 30 sec	72°C, 30 sec	72°C, 10 min	4°C ≅
		35 cycles				
**MLST**	94°C, 2 min	94°C, 30 sec	A*, 45 sec	72°C, 1.5 min	72°C, 10 min	4°C ≅
		36 cycles				

A* Annealing temperature for primer *hcp*A - 53°C, *fts*Z and *gat*B - 54°C, *cox*A - 55°C, *fbp*A - 59°C
